# Near Infrared‐Mediated Intracellular NADH Delivery Strengthens Mitochondrial Function and Stability in Muscle Dysfunction Model

**DOI:** 10.1002/advs.202415303

**Published:** 2025-01-31

**Authors:** Hui Bang Cho, Hye‐Ryoung Kim, Sujeong Lee, Chae Won Cho, Ji‐In Park, Seulki Youn, Gyuwon So, Sumin Kang, Hye Jin Kim, Keun‐Hong Park

**Affiliations:** ^1^ Department of Nano‐regenerative Medical Engineering College of Life Science CHA University 6F, CHA Biocomplex, Sampyeong‐Dong, Bundang‐gu Seongnam‐si 13488 Republic of Korea

**Keywords:** mitotherapy, mitochondrial priming, muscle regeneration, NADH delivery, near‐infrared (NIR)

## Abstract

Mitochondrial transfer emerges as a promising therapy for the restoration of mitochondrial function in damaged cells, mainly due to its limited immunogenicity. However, isolated mitochondria rapidly lose function because they produce little energy outside cells. Therefore, this study investigates whether near infrared (NIR)‐mediated nicotinamide adenine dinucleotide (NADH) pre‐treatment enhances mitochondrial function and stability in mitochondria‐donor cells prior to transplantation. Clinical applications of NADH, an essential electron donor in the oxidative phosphorylation process, are restricted due to the limited cellular uptake of NADH. To address this, a photo‐mediated method optimizes direct NADH delivery into cells and increases NADH absorption. L6 cells treated with NADH and irradiated with NIR enhanced NADH uptake, significantly improving mitochondrial energy production and function. Importantly, the improved functional characteristics of the mitochondria are maintained even after isolation from cells. Primed mitochondria, i.e., those enhanced by NIR‐mediated NADH uptake (P‐MT), are encapsulated in fusogenic liposomes and delivered into muscle cells with mitochondrial dysfunction. Compared to conventional mitochondria, P‐MT mitochondria promote greater mitochondrial recovery and muscle regeneration. These findings suggest that NIR‐mediated NADH delivery is an effective strategy for improving mitochondrial function, and has the potential to lead to novel treatments for mitochondrial disorders and muscle degeneration.

## Graphical abstract

A therapeutic approach to address muscle dysfunction caused by mitochondrial dysfunction. Near‐infrared irradiation is used to induce nicotinamide adenine dinucleotide (NADH) uptake in healthy muscle cells, enhancing the structural and physiological properties of their mitochondria. These strengthened mitochondria are transplanted into damaged muscle cells to improve muscle dysfunction.

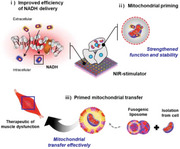

## 1 Introduction

Mitochondrial dysfunction plays a critical role in muscle dysfunction, particularly in conditions characterized by the progressive loss of muscle mass and strength, such as sarcopenia and muscle atrophy.^[^ ^1^ ^]^ Within muscle cells, mitochondria are essential for energy metabolism, adenosine triphosphate (ATP) production, regulation of reactive oxygen species (ROS), and maintenance of intracellular calcium homeostasis.^[^ ^2^ ^]^ When mitochondrial function declines, the ability to produce ATP diminishes, leaving muscle fibers with an inadequate energy supply.^[^ ^3^ ^]^ This leads to weakened muscle contraction and impaired recovery. Additionally, mitochondrial dysfunction increases ROS production, causing oxidative stress, which in turn accelerates muscle cell damage and apoptosis.^[^ ^4^ ^]^ Mitotherapy, the transfer of healthy mitochondria from donor to damaged cells or tissues, is receiving increasing attention as an innovative approach for the restoration of mitochondrial function and cellular energy metabolism in damaged cells.^[^ ^5^ ^]^ Although mitotherapy has high therapeutic potential, several difficulties, such as structural and functional damage during the mitochondrial isolation and transplantation process, limit its clinical application.^[^ ^6^ ^]^ Moreover, because mitochondria produce ATP through oxidative phosphorylation, they require a continuous supply of essential metabolites and cofactors.^[^ ^7^ ^]^ However, when mitochondria are isolated from the cellular environment, this supply is cut off, and their function can rapidly decline.^[^ ^8^ ^]^ Therefore, although mitotherapy is promising, ensuring that mitochondria survive and function outside their native cellular environment remains a major challenge.

To address these challenges and improve mitochondrial function, we investigated whether the direct delivery of nicotinamide adenine dinucleotide (NADH) into mitochondria would improve their survival and function after their separation from their host cells.^[^ ^9^ ^]^ NADH is an essential cofactor involved in numerous biochemical reactions and serves as a primary electron donor in the mitochondrial electron transport chain, which drives oxidative phosphorylation and ATP synthesis.^[^ ^10^ ^]^ In particular, NADH donates electrons to complex I (NADH dehydrogenase), the first and main entry point of the electron transport chain, making it vital for maintaining mitochondrial energy production.^[^ ^11^ ^]^ However, NADH is a hydrophilic molecule with a negative charge, which impedes its transfer across the hydrophobic lipid bilayer of the cell membrane and delivery into mitochondria.^[^ ^12^ ^]^ This limitation restricts the therapeutic potential of using NADH to enhance mitochondrial function.

In this study, we describe an NADH delivery strategy that employs light‐emitting diode (LED)‐mediated changes in the cell membrane to increase the delivery of NADH into cells, which has been validated in previous studies. We investigated how LEDs, especially at NIR wavelengths, induce pore formation in artificial lipid membranes composed of phosphatidylcholine (PC), phosphatidylethanolamine (PE), and a binary mixture of PC/PE. Our findings show that NIR irradiation induces the deprotonation of the ammonium ion (NH₃⁺) at the terminus of the PE headgroup, converting it into an amine group (NH₂).^[^ ^13^ ^]^ The NIR wavelength in LED selectively alters the permeability of the cell membrane to allow for efficient intracellular delivery of molecules such as NADH.

As previously discussed, to address the limitations of mitotherapy, we propose a method to deliver NADH to cells, inducing mitochondrial priming to facilitate the replacement of damaged mitochondria. This study further employs fusogenic liposomes to enable the efficient transfer of primed mitochondria into cells associated with muscle dysfunction.^[^ ^14^ ^]^

In summary, this study seeks to develop an innovative strategy for the restoration of mitochondrial function in degenerative diseases that relies on combining NIR irradiation‐based NADH delivery to prime mitochondria with fusogenic liposome‐mediated mitochondrial transfer.

## 2 Results and Discussion

### 2.1 NIR Irradiation Enhances NADH Uptake and Boosts Mitochondrial Function in L6 Cells

**Figure 1:**
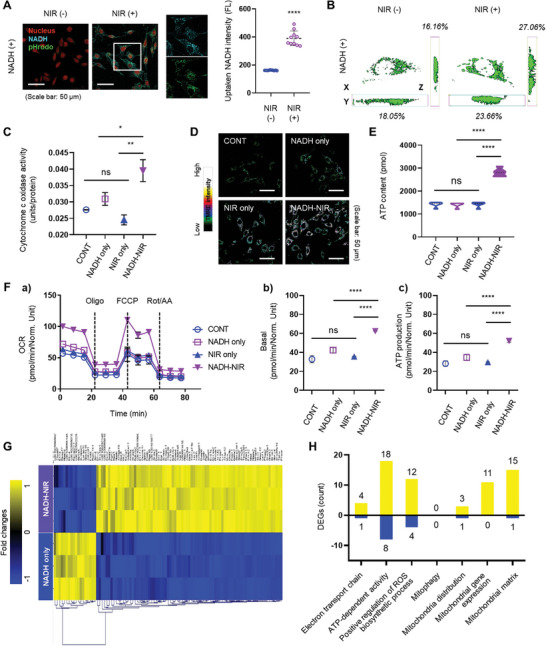
NIR‐mediated NADH delivery and mitochondrial priming in L6 cells. A) Confocal laser scanning microscopy images and quantitative graphs showing NADH (cyan) and pH changes (green), Nucleus (red) in L6 cells after LED irradiation (*n* = 3). Scale bar = 50 µm. B) Quantitative analysis of mitochondrial density using CLSM of 10‐N‐nonyl acridine orange (NAO) stained samples. C) Assessment of cytochrome c oxidase (CCO) activity (*n* = 3). TMRE staining images for the assessment of mitochondrial membrane potential (Δψm) Scale bar = 50 µm D) and ATP content in cells after NIR‐mediated NADH delivery E) (*n* = 3). F) Seahorse analysis showing mitochondrial activities after NIR‐mediated NADH delivery (*n* = 3). G) Heap map analysis of mitochondrial‐related gene expression patterns between NADH only (*n* = 3, blue) and NADH‐NIR (*n* = 3, purple). Yellow indicates up‐regulation and blue indicate down‐regulation. DEGs were defined by a *p*‐value < 0.05 and |log2FC| >1. H) Graph of the number of DEGs according to the GO terms of mitochondria‐related genes. Yellow indicates up‐regulation and blue indicate down‐regulation. Data represent the mean ± SD. Statistical significance was evaluated using one‐way ANOVA with Tukey's multiple comparisons test. ns, not significant. ^*^ *p* < 0.05, ^**^ *p* < 0.01, and ^****^ *p* < 0.0001.

This study focuses on enhancing mitochondrial function by promoting the uptake of NADH through changes in the cell membrane induced by NIR irradiation,^[^ ^13a–c^ ^]^ utilizing the electron transport system via redox reactions. L6 cells were treated with 20 µm NADH and subjected to NIR irradiation. After exposure to NIR light, cyan fluorescence indicating the presence of NADH inside the cells and pH changes due to the generation of H+ ions (shown in green) were observed. These results suggest that NIR irradiation promotes the intracellular delivery of NADH, leading to the production of NAD+ and H+ through redox reactions (**Figure** **1**A).

To determine the optimal NADH concentration and NIR irradiation time, L6 cells were treated with NADH at concentrations of 0, 1, 10, 20, and 30 µm and then exposed or not to NIR irradiation. In the NADH‐treated cells exposed to NIR irradiation (NADH‐NIR group), the amount of intracellular NADH increased in proportion to the NADH concentration, with a maximum observed at 20 µm. The intracellular level of NAD+ also increased, which is suspected to have been due to the increased amounts of NAD+ generated by NADH dehydrogenase. By contrast, the intracellular levels of NADH and NAD+ in NADH‐treated cells not exposed to NIR irradiation (NADH‐only group) were very low or undetectable (Figure S1, Supporting Information). To further refine the treatment duration, we employed a fixed concentration of NADH (20 µm) and measured the oxygen consumption rate (OCR) at 0, 2, 4, and 6 h post‐NIR irradiation (Figure S2A, Supporting Information). In the NADH‐only group, there was no increase in OCR, because NADH could not enter the cells. However, in the NADH‐NIR group, NADH successfully entered the cells, leading to an increase in OCR levels, particularly at 2 and 4 h after NIR irradiation. After 6 h, a noticeable decrease in OCR was observed, confirming that the optimal NIR irradiation duration for increasing mitochondrial function was 4 h (Figure S2B, Supporting Information).

To confirm that NADH was effectively delivered into mitochondria and initiated mitochondrial priming, complex I—the primary site of NADH oxidation—was inhibited with rotenone. In the absence of rotenone, priming would occur if NADH reached the mitochondria, whereas in the presence of rotenone, priming would be prevented. The results showed that priming in the NADH‐NIR group occurred in the absence of rotenone, but not in the presence of rotenone. This confirms that NADH delivered into the cell via NIR irradiation is oxidized by mitochondrial complex I, leading to mitochondrial priming (Figure S3, Supporting Information).

To visually confirm that mitochondrial were primed by NADH uptake, intracellular mitochondrial density was measured using 10‐N‐nonyl acridine orange (NAO) (Figure 1B), which selectively binds to cardiolipin, a constituent of the mitochondrial inner membrane. This provides a method that can be used to measure specifically mitochondrial quantity and quality. The results showed that the volume of the NAO signal was higher in the NADH‐NIR group than in the NADH‐only group.

To further analyze the effect of NADH and NIR treatment on mitochondria activity, we measured the activity of cytochrome C oxidase (CCO), which is the final complex (complex IV) in the electron transport chain (ETC) that is responsible for energy production in the mitochondrial inner membrane. While no significant differences were observed between the NADH‐only and NIR‐only groups relative to the control group (CONT), the NADH‐NIR group showed a significant increase in CCO activity (Figure 1C).

Additionally, we measured mitochondrial membrane potential (MMP) using tetramethylrhodamine, ethyl ester (TMRE), which selectively accumulates in the mitochondrial inner membrane. An increase in TMRE intensity indicates an increase in MMP, suggesting that the cells are producing energy more efficiently. Compared with the CONT group, the TMRE signal intensities of the NADH‐only and NIR‐only groups increased, but the increases were inferior to that of NADH‐NIR group (Figure 1D). We verified whether the increase in CCO activity and MMP was connected to ATP production (Figure 1E). The results showed that the ATP content of the NADH‐NIR group was approximately two‐fold higher than those of the other groups and that the increase in ATP content was proportionate to the increase in NADH concentration. (Figure S4, Supporting Information).

We next evaluated mitochondrial respiration and activity using the Seahorse assay. OCR, a critical indicator of mitochondrial metabolic activity and energy production, was significantly higher in the NADH‐NIR group than in the other groups, suggesting cell metabolic activity was increased by NIR irradiation‐mediated uptake of NADH. The increased metabolic activity led to functional changes in the cells, including higher basal respiration and ATP production. Basal respiration reflects the oxygen consumption required by cells to sustain their basic energy needs, while increased ATP production signifies that the cells can meet their energy demands (Figure 1F).

We next verified the priming of mitochondria in the NADH‐NIR group and explored the changes occurring at the transcriptomic level using differential expression (DE) analysis. First, a PCA plot was used to visualize similarities in transcriptome expression between groups. The CONT and NIR‐only groups showed very similar transcriptome expression profiles, which were also similar to that of the NADH‐only group. By contrast, the NADH‐NIR group showed significant differences in transcriptome expression compared with the other groups. A volcano plot confirmed the significant differences in transcription expression between the NADH group and NADH‐NIR group (Figure S5A,B, Supporting Information). To identify differential expression genes (DEGs), we compared the genes expressed in the NADH‐NIR group with those expressed in the NADH‐only group and then filtered and visualized the DEGs using a heat map graph (Figure 1G). Many more genes were upregulated (yellow) in the NADH‐NIR group than in the NADH group, while by comparison, few genes were downregulated (blue). GO enrichment analysis of the DEGs revealed that some upregulated genes belonged to categories related to the ETC and ATP‐dependent activity, confirming the increase in qualitative mitochondrial priming in the NADH‐NIR group. Additionally, numerous genes related to mitochondrial distribution, genes, and matrix were also differentially expressed, suggesting quantitative mitochondrial priming had occurred in the NADH‐NIR group. No DEGs corresponding to mitophagy‐related genes were observed, confirming the above interpretation (Figure 1H). To explore the molecular mechanism through which NIR irradiation enhances intracellular NADH uptake and induces mitochondrial priming, we performed KEGG pathway analysis based on the DEG results shown in Figure 1G,H. This analysis identified biological pathways significantly activated by NADH uptake facilitated by NIR irradiation, with strong enrichment in pathways related to mitochondrial function and energy metabolism. The key pathway identified was the MAPK signaling pathway, which upregulates the expression of PGC1α, a key regulator of mitochondrial function. This pathway promotes mitochondrial biogenesis and enhances antioxidant defense mechanisms^[^ ^15^ ^]^ (Figure S5C, Supporting Information). qRT‐PCR was performed to determine whether the MAPK signaling pathway was involved in mitochondrial priming. The analysis revealed that PGC1α levels increased by approximately 2‐fold in the NADH‐NIR group, while no significant changes were observed in the NADH‐only or NIR‐only groups. These results confirm that NADH delivered into cells via NIR irradiation activates the MAPK signaling pathway, effectively inducing mitochondrial priming (Figure S5D, Supporting Information). Beyond mitochondria, the expression of genes involved in skeletal system development, angiogenesis, and cell migration was also increased in the NADH‐NIR group (Figure S5E, Supporting Information).

### 2.2 Primed Mitochondria Retain Structural, Genetic, and Functional Stability After Isolation

To confirm that the mitochondria in cells treated with NADH‐NIR retained priming after their isolation from cells, we examined the CCO, TMRE, and ATP levels of the mitochondria isolated from cells that had been treated for different times with NADH‐NIR. The results showed that an incubation time of 4 h resulted in isolated mitochondria with increases in CCO, TMRE, and ATP levels that were similar to those of the mitochondria in cells incubated with NADH‐NIR for the same time, demonstrating that a 4 h incubation time is optimal for obtaining isolated primed mitochondria (Figure S6, Supporting Information).

**Figure 2:**
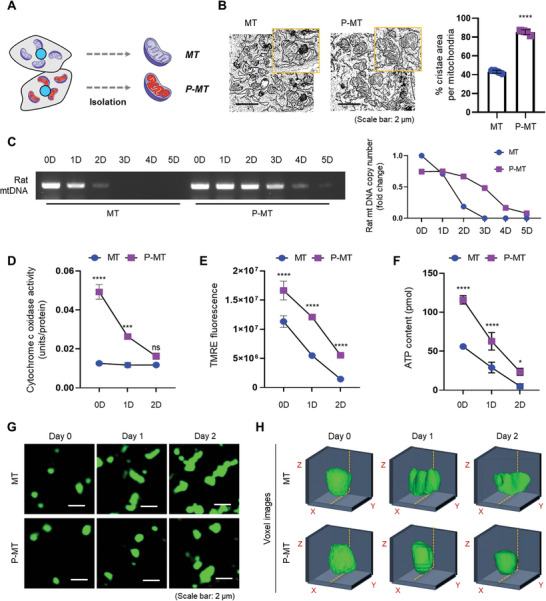
Comparative analysis of MT and P‐MT isolated from L6 cells. A) Schematic illustration of experimental grouping for analyzing isolated MT and P‐MT. B) TEM images showing mitochondrial cristae density (*n* = 4). Scale bar = 2 µm. The graph shows % cristae area per mitochondrion. C) mtDNA copy number analysis to compare the lifespans of MT and P‐MT. D) Functional analysis of CCO (*n* = 3). E) Mitochondrial membrane potential (Δψm) via TMRE fluorescence (*n* = 3). F) ATP levels in MT and P‐MT up to 2 days post‐isolation (*n* = 3). Confocal laser scanning microscopy images G) and voxel view H) comparing the mitochondrial morphological stability up to 2 days post‐isolation. Data represent the mean ± SD. Statistical significance was evaluated using one‐way ANOVA with Tukey's multiple comparisons tests. ns, not significant. ^*^ *p* < 0.05, ^***^ *p* < 0.001, and ^****^ *p* < 0.0001.

A key factor for effective mitotherapy is maintaining the original structure and activity of isolated mitochondria. To assess these properties of the isolated mitochondria, we isolated mitochondria from L6 cells treated with NADH in the presence or absence of NIR irradiation. The mitochondria isolated from cells that were not exposed to NIR irradiation were labeled as MT, while those isolated from cells that were exposed to NIR irradiation were labeled as P‐MT (**Figure** **2**A).

Mitochondria possess a smooth outer membrane and a folded inner membrane that forms cristae; it is known that the greater the cristae density, the more efficient energy production becomes.^[^ ^16^ ^]^ Transmission electron microscopy (TEM) imaging results showed that P‐MTs had approximately twice the cristae area of that of MTs, indicating that they produced energy more efficiently than MTs (Figure 2B).

To compare the biological stability of MTs and P‐MTs, we measured their lifespan using mtDNA copy number analysis. Although the amount of mtDNA on day 0 was similar between MTs and P‐MTs, mtDNA in MTs began to decrease from day 1 and had disappeared by day 3, whereas the mtDNA of P‐MT remained stable until day 5 (Figure 2C). These results suggest that NADH‐NIR treatment helps maintain the genetic stability of isolated mitochondria for a longer period than with NADH treatment alone, which can be interpreted as evidence that NADH‐NIR treatment preserves the vitality of isolated mitochondria.

Next, we examined the changes in CCO, TMRE, and ATP levels of MTs and P‐MTs up to 2 days after their isolation to evaluate time‐dependent changes in their priming. P‐MTs on day 0 exhibited high CCO activity and decreased activity on day 1, but the activity on day 1 remained higher than that of MTs. By day 2, the CCO activity of both groups showed a similar reduction. By contrast, MTs displayed minimal activity on day 0, indicating reduced stability post‐isolation (Figure 2D). Both TMRE and ATP levels (Figure 2E,F) remained consistently higher in P‐MTs than in MTs until day 2, suggesting that P‐MTs are more effective at maintaining their function and exhibit greater functional stability. Finally, the structural stability of MTs and P‐MTs was assessed up to 2 days post‐isolation. MTs showed aggregation or structural breakdown after day 1, whereas P‐MTs retained their structural stability up to day 2 (Figure 2G,H). Overall, these results demonstrate that P‐MTs are superior to MTs with respect to the preservation of mitochondrial structural and biogenetic stability.

### 2.3 P‐MTs Loaded Fusogenic Liposomes Reduce Mitochondrial Stress in a 2D Model of LPS/Rot‐induced Muscle Dysfunction

**Figure 3:**
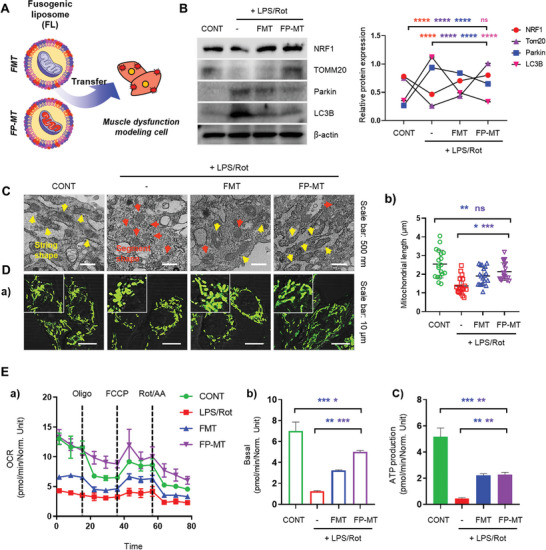
Comparative of mitochondrial function restoration in a muscle dysfunction cell model using FMT and FP‐MT. A) Schematic illustration of FL encapsulation and delivery of MT and P‐MT into a muscle dysfunction cell model. B) Western blot analysis of mitochondrial biogenesis markers (NRF1, Tom20) and mitophagy markers (Parkin, LC3B) after treatment with FMT and FP‐MT (*n* = 3). C) Mitochondrial morphological changes observed in TEM images. The yellow arrow indicates string shape and the red arrow indicates the segment shape. Scale bar = 500 nm. D) Confocal microscopy images of Mitotracker fluorescence analysis showing mitochondrial morphology and distribution (a) with quantitative data (b) (*n* = 19). Scale bars D, a = 10 µm. E) Seahorse assay showing differences in cell respiration (a), with basal respiration (b) and ATP production (c) displayed in a quantitative graph (*n* = 3). Data represent the mean ± SD. Statistical significance was evaluated using one‐way ANOVA with Tukey's multiple comparisons tests. ns, not significant. ^*^ *p* < 0.05, ^**^ *p* < 0.01, ^***^ *p* < 0.001, and ^****^ *p* < 0.0001.

Next, we utilized fusogenic liposomes (FLs), a technology developed by our research team,^[^ ^14^ ^]^ to deliver P‐MTs into cells with the aim of restoring muscle dysfunction (**Figure** **3**A). To confirm that MTs and P‐MTs were encapsulated within FLs, we measured changes in liposome size and zeta potential using dynamic light scattering (DLS) (Figure S7A,B, Supporting Information). In the encapsulation tests, we refer to FL‐encapsulated MTs as FMT and the FL‐encapsulated P‐MTs as FP‐MT. The size measurements indirectly confirmed that both FMT and FP‐MT were encapsulated at a 1:1 ratio. MT and P‐MT have a negative charge of ≈−30 mV, while the positively charged lipids in the liposomes have a high charge of over +40 mV. After encapsulation, positive charges were detected, confirming successful encapsulation.

We used confocal laser scanning microscopy (CLSM) to visually confirm that MTs and P‐MTs were encapsulated within the FLs. MTs and P‐MTs labeled with Mitotracker Green were successfully encapsulated within the liposomes, and the green fluorescence overlapped with the rhodamine red fluorescence of the liposomes. These overlapping yellow signals confirmed that MTs and P‐MTs were successfully encapsulated within the liposomes, and voxel images showed that MTs and P‐MTs remained in a stable state within the liposomes (Figure S7C, Supporting Information). To confirm the internalization pattern of FMT and FP‐MT, we used specific dyes: the green signal represents the plasma membrane (PM), the red signal represents the FL, and the blue signal represents the transferred MT. In cells exposed to FL, the overlap of green and red signals indicated fusion between FL and the PM (yellow), and both MT and P‐MT were observed in the cytosol for FMT and FP‐MT (blue). This suggests, as demonstrated in previous studies, that MTs and P‐MTs encapsulated by FLs are delivered through both endocytosis and membrane fusion pathways (Figure S8A, Supporting Information). The internalization rate of FLs through the membrane fusion pathway was 98% for both FMT and FP‐MT (Figure S8B, Supporting Information). Finally, to quantitatively verify this process, we analyzed mtDNA copy number after delivering heterologous MTs and P‐MTs. Since the donor MTs were derived from a rat myoblast L6 cell line and the recipient cells were human‐derived mesenchymal stem cells (MSCs, xenogeneic), we could accurately track delivery efficiency. Both FMT and FP‐MT delivered more mitochondria to cells than MTs and P‐MTs, with no difference in the amount of MT and P‐MT delivered (Figure S8C, Supporting Information). Therefore, we used FLs to ensure stable and efficient delivery of mitochondria into cells.

To establish a reliable muscle dysfunction model, various concentrations of lipopolysaccharide (LPS) and rotenone (Rot) were employed to determine their non‐toxic levels (Figure S9A, Supporting Information). The optimal concentrations within the non‐toxic range were identified by performing NAD+/NADH assays and qRT‐PCR analysis. Intracellular levels of NAD+/NADH and key muscle‐related markers, including MURF‐1 (a marker of muscle protein degradation) and MyoD (associated with skeletal muscle development and myogenesis) were assessed. Based on the results of these assays, we selected 1 µg mL^−1^ LPS and 0.1 µm rotenone as the optimal concentrations that did cause cell toxicity (Figure S9B,C, Supporting Information).

Next, we evaluated whether mitochondrial stress, which is responsible for muscle dysfunction, would be restored in the cells of a 2D muscle dysfunction model after FL‐mediated delivery of P‐MT. After delivery of FMT and FP‐MT to cells, we found that the expression of mitochondrial biogenesis markers NRF1 and Tom20 was reduced in the LPS/Rot‐treated group, slightly increased in the FMT group, and restored to levels similar to those of the CONT in the FP‐MT group. Notably, Tom20 expression was higher than the control level in the FP‐MT group, indicating a strong recovery response. To further evaluate the fate of mitochondria transferred into cells, we analyzed the expression of mitophagy markers Parkin and LC3B, which are involved in the mitochondrial degradation process. The expression levels of both markers were significantly lower in the FP‐MT group than in the FMT group (Figure 3B).

And we evaluated mitochondrial structure and distribution to follow recovery from LPS/Rot‐induced muscle dysfunction. Cell‐TEM (Figure 3C) and Mitotracker fluorescence analysis (Figure 3D) revealed that mitochondria in the LPS/Rot‐treated group were significantly damaged and fragmented into small, balloon‐like shapes (red arrow). Mitochondria in the FMT showed partial restoration of about half of the mitochondria to a string‐like structure, similar to that of the CONT group (yellow arrow). However, mitochondria in the FP‐MT‐treated group recovered substantially, as evidenced by their longer, interconnected morphology.^[^ ^17^ ^]^

Finally, mitochondrial OCR was measured. Basal respiration was noticeably higher in the FP‐MT group than in the FMT treatment group. ATP production, a direct indicator of mitochondrial energy‐generating capacity, recovered to similar levels in both the FMT and FP‐MT treatment groups. Cells typically balance ATP production with cellular energy requirements, so even with the increased respiration in FP‐MT‐treated cells, ATP production may be self‐regulated to meet cellular demand without being overproduced (Figure 3E).

Additionally, to assess skeletal muscle development, myogenesis, and mitochondrial stress, we analyzed the expression of Myogenin and MyoD. In the LPS/Rot‐treated group, the expression of these markers was reduced, while their expression was significantly increased in the FP‐MT‐treated group and exceeded those in the FMT‐treated group, which showed near‐CONT levels of these markers. These findings indicate that FP‐MT treatment not only restores mitochondrial stress more effectively than FMT treatment but also increases the expression of key muscle development markers to a greater extent, demonstrating its superior potential for the treatment of muscle dysfunction. (Figure S10, Supporting Information).

FP‐MT was more effective than FMT in increasing mitochondrial biogenesis, improving mitochondrial structural recovery, and increasing the expression of muscle development markers. These results suggest that FP‐MT may provide a robust approach for the reversal of muscle dysfunction associated with mitochondrial stress.

### 2.4 FP‐MT Transplantation Enhances Recovery and Promotes Early Differentiation in a Bioengineered 3D Skeletal Muscle Model of Dysfunction

**Figure 4:**
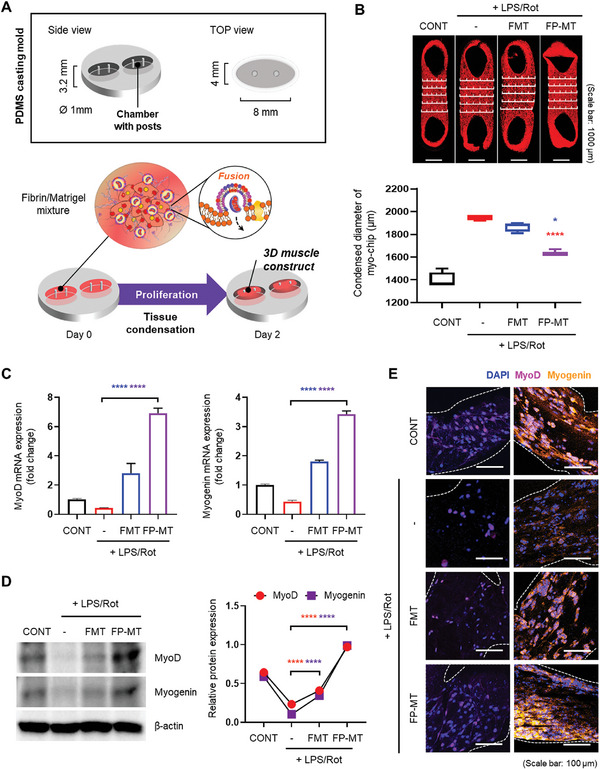
Assessment of muscle recovery and early differentiation in a 3D muscle construct after FMT and FP‐MT transplantation. A) Schematic illustration of the PDMS casting mold and Fibrin/Matrigel matrix embedded with muscle dysfunction cells and FMT or FP‐MT. Following a 2‐day proliferation period, cell recovery was assessed. B) Confocal laser scanning microscopy imaging of TRIRC‐stained Fibrin/Matrigel matrix and comparison of condensation. Scale bar = 1000 µm. qRT‐PCR C) and Western blot analysis D) showing expression levels of myogenesis‐related markers (*n* = 3). E) Immunostaining analysis of myogenic differentiation protein. Scale bar = 100 µm. Data represent the mean ± SD. Statistical significance was evaluated using one‐way ANOVA with Tukey's multiple comparisons tests. ns, not significant. ^*^ *p* < 0.05, and ^****^ *p* < 0.0001.

The effectiveness of FP‐MT in recovering muscle dysfunction was tested in a 3D Fibrin/Matrigel matrix that closely mimics the structure and function of muscle tissue.^[^ ^18^ ^]^ Damaged muscle cells, along with FMT or FP‐MT, were embedded together in the matrix and placed within a manufactured mold. Following a 2‐day proliferation period, recovery was examined. The mold was made of PDMS and the matrix consisted of Fibrin and Matrigel (**Figure** **4**A).

CLSM imaging of the TRIRC‐stained Fibrin/Matrigel confirmed that cells that did not recover from LPS/Rot‐induced damage had a long horizontal length in the Myo chip, which is attributed to a lack of condensation. By contrast, damaged muscle cells exposed to FP‐MT in the matrix had shorter lengths than those exposed to FMT, indicating greater recovery and increased condensation (Figure 4B).

To further examine the mechanisms underlying improved contraction and relaxation of muscle cells following FP‐MT exposure in an early muscle injury model, we performed qRT‐PCR analysis to assess the expression of MyoD and Myogenin, which are essential markers of early muscle development. The results showed that their expression in the LPS/Rot‐exposed group decreased, whereas their expression in both the FMT‐ and FP‐MT‐exposed groups increased substantially. Notably, their expression in the FP‐MT‐exposed group was significantly higher than in both the CONT group and FMT‐exposed group. This suggests that FP‐MT‐mediated recovery of damaged muscle cells involves an active regenerative process, with MyoD and Myogenin expression exceeding normal levels, indicating damage repair (Figure 4C).

To confirm these findings at the protein level, we performed a Western blot analysis of MyoD and Myogenin expression. Consistent with the genomic results, protein levels of MyoD and Myogenin were reduced in the LPS/Rot‐exposed group, whereas they were significantly increased in the FP‐MT‐exposed group to levels that were superior to those of the CONT and FMT‐exposed groups. MyoD and Myogenin protein expression in the FMT‐exposed group was comparable to that in CONT group (Figure 4D), which is different from the results of qRT‐PCR. Thus, FMT‐induced increases in the mRNA levels of Myogenin and MyoD may not translate into a corresponding increase in the protein levels of these markers. By contrast, FP‐MT exposure effectively boosted both mRNA and protein expression levels, demonstrating its superior impact on muscle recovery and regeneration processes.

Finally, the expression of MyoD and Myogenin was further validated by fluorescent immunostaining of cells within the hydrogel matrix. The results were similar to those of Western blot analysis (Figure 4E).

In conclusion, these results demonstrate that FP‐MT significantly increases muscle recovery and early differentiation in a 3D mitochondrial transplantation model.

### 2.5 FP‐MT Treatment Enhances Myogenesis and Skeletal Muscle Development Compared to FMT in Differentiation Model

**Figure 5:**
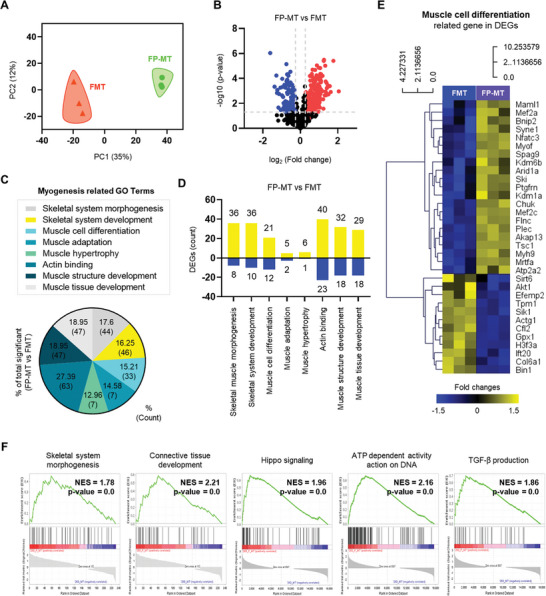
Transcriptomic analysis of FMT versus FP‐MT in 3D muscle constructs. A) PCA plot showing distinct transcript expression patterns between FMT (*n* = 3) and FP‐MT (*n* = 3) groups after 26 days of differentiation. B) Volcano plot highlighting differences in gene expression between FMT and FP‐MT‐exposed constructs. Red indicates up‐regulation and blue indicates down‐regulation. C) DEGs filtered by myogenesis‐related GO terms, visualized in a graph. DEGs were defined by |log2FC| >  1 and *p*‐value < 0.05. D) Comparison of upregulated (yellow) and downregulated (blue) genes in FP‐MT versus FMT‐exposed constructs. E) Heatmap of DEGs related to muscle cell differentiation, showing the distinct expression profiles of FMT (*n* = 3) and FP‐MT‐exposed constructs (*n* = 3). F) GSEA analysis of myogenic differentiation pathway‐related genes in FP‐MT‐exposed constructs.

In previous experiments, we confirmed that both FMT and FP‐MT effectively promoted early differentiation. Therefore, DE analysis was conducted after differentiation with FMT and FP‐MT constructs for up to 26 days. First, PCA plots were used to visualize similarities in transcript expression between groups. The results showed that FMT‐ and FP‐MT‐exposed groups had significantly different transcript expression patterns (**Figure** **5**A). This difference was further confirmed by the volcano plot, which demonstrated substantial expression differences between the two groups (Figure 5B). DEGs were filtered by myogenesis‐related GO terms and displayed in a graph (Figure 5C). Compared with the FMT‐exposed group, more genes were upregulated (yellow) and fewer genes were downregulated (blue) in FP‐MT‐exposed group (Figure 5D). GO analysis of these DEGs showed that the upregulated genes were primarily associated with myogenesis, which is in agreement with the recovery and early differentiation demonstrated by the previous results above. Additionally, several genes related to actin binding and muscle structure development were identified, which may indicate further recovery and structural organization. Based on these findings, DEGs related to muscle cell differentiation were visualized in a heatmap, demonstrating the distinct expression profiles of the FMT‐ and FP‐MT‐exposed groups (Figure 5E). Gene set enrichment analysis (GSEA) also revealed that skeletal system morphogenesis, connective tissue development, Hippo signaling, ATP‐dependent activity action on DNA, and TGF‐β production pathways were significantly upregulated in the FP‐MT group (Figure 5F).

To further examine the differences in the promotion of skeletal muscle development between FMT and FP‐MT, we conducted a comparative DE analysis of FMT‐exposed samples and the LPS/Rot‐exposed control sample, as well as a comparative DE analysis of FP‐MT‐exposed samples and the LPS/Rot‐exposed control sample. The results showed minimal differences between the FMT‐exposed samples and the LPS/Rot‐exposed control sample, whereas the FP‐MT‐treated samples showed substantial differences relative to the LPS/Rot‐exposed control sample.

In the Gene Ontology Biological Process (GO‐BP) analysis, the processes enriched in the FMT‐exposed samples versus LPS/Rot‐exposed control sample were largely unrelated to skeletal development, including processes such as Negative Regulation of Apoptotic Process, Positive Regulation of Gene Expression, and Cell Migration, among others. By contrast, in the FP‐MT‐treated samples versus the LPS/Rot‐exposed control sample, numerous processes were closely linked to skeletal development, including Actin Cytoskeleton Organization, Skeletal System Morphogenesis, Myoblast Differentiation, and Skeletal System Development (Figure S11, Supporting Information).

These results suggest that, unlike FMT‐activated pathways, FP‐MT activated pathways and processes were directly associated with skeletal muscle formation and structural organization over an extended period of 26 days, further reinforcing the superiority of FP‐MT for the promotion of myogenesis and skeletal tissue development.

### 2.6 FP‐MT Muscle Constructs Enhance Myotube Formation, Maturation, and Contractile Function, Showing Improved Recovery Compared to FMT

**Figure 6:**
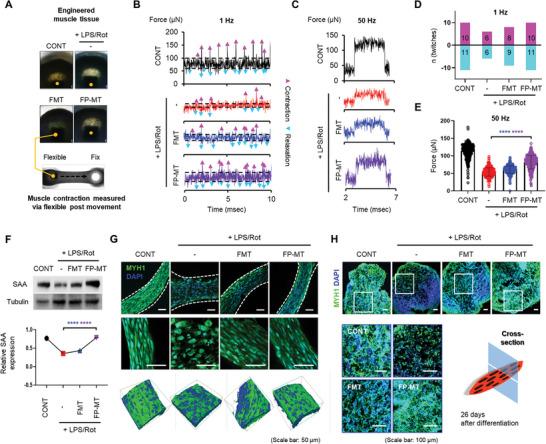
Myomere formation and contractile function assessment of FMT and FP‐MT transplanted muscle constructs. A) Contractility assessment after 26 days of differentiation. Photograph of the setup for measuring the contractile forces of muscle constructs. Representative twitch (1 Hz) B) and tetanic (50 Hz) force C) traces of constructs. D) Contraction and relaxation twitches at 1 Hz E) and tetanic forces graph at 50 Hz are based on 400 points collected from 50 Hz tetanic stimulation. F) Western blot analysis of SAA expression in constructs (*n* = 3). G) MYH1 expression and 3D voxel imaging reveal multinuclear myofiber bundles. Scale bar = 50 µm. H) Cross‐sectional analysis indicating superior muscle fiber recovery. Scale bar = 100 µm. Data represent the mean ± SD. Statistical significance was evaluated using one‐way ANOVA with Tukey's multiple comparisons tests. ^****^ *p* < 0.0001.

Skeletal muscle contractility is one of the key indicators of skeletal muscle health and strength. To assess differences in contractility, we measured the contractility of muscle structures after electrical stimulation. After 26 days of differentiation, all groups produced twitch and tetanic contractions in response to electrical stimulation of 1 and 50 Hz, respectively (**Figure** **6**A). The 50 Hz frequency showing a tetanic force in all samples was determined through preliminary screening (Figure S12, Supporting Information). In the twitch force (1 Hz) graph, analysis of the amplitude of the force generated by each group (black dotted line) showed that the amplitude generated by the FP‐MT‐exposed group was similar to that of the CONT and higher than that of the FMT‐exposed group (Figure 6B). Additionally, quantitative analysis of the number of contractions and relaxations at 1 Hz revealed that the unexposed CONT group and FP‐MT‐exposed produced 21 contractions and relaxation, whereas the LPS/Rot‐exposed group produced 12 contractions and MT‐exposed group 17 contractions (Figure 6D). At 50 Hz (Figure 6C), the unexposed CONT and FP‐MT‐exposed group exhibited a tetanic force ≈150 µm of construction, whereas the LPS/Rot‐exposed group produced a tetanic force at 60 µm and MT at 80 µm (Figure 6E). Thus, the P‐MT‐exposed constructs exhibited superior twitch and tetanic forces compared with the FMT‐ and LPS/Rot‐exposed constructs.

Expression of sarcomeric α‐actinin (SAA) was analyzed to assess the maturity of muscle structures after 26 days of differentiation. The level of SAA expression in the FP‐MT‐treated group was similar to that of the CONT group, while the FMT‐treated group showed SAA expression levels comparable to the LPS/Rot‐treated group, which showed minimal SAA expression (Figure 6F).

To evaluate whether FP‐MTs increase muscle differentiation and myotube maturation, we analyzed differences in myofiber bundles by measuring MYH1 expression. Consistent with previous findings, muscle fiber bundle recovery in the FP‐MT‐exposed constructs reached levels comparable to that of the CONT. Although FMT‐exposed contracts also showed an increase in fiber bundles compared with the LPS/Rot‐exposed constructs, the level of recovery was significantly greater in the P‐MT‐exposed constructs. Additionally, through 3D voxel imaging, we observed more multinuclear structures within muscle fibers in the FP‐MT‐exposed constructs than in the FMT‐exposed constructs (Figure 6G). Additional analysis of the cross‐sections of myobundles showed that the FP‐MT‐exposed constructs had a higher level of muscle fiber recovery than the FMT‐exposed constructs. (Figure 6H).

In summary, P‐MT‐exposed muscle constructs had improved muscle differentiation, maturation, and contractile function, achieved recovery levels similar to the controls, and outperformed the recovery of MT‐exposed muscle constructs.

## 3 Conclusion

This study highlights the potential of NIR‐mediated NADH delivery as a novel mitotherapy‐based approach to treat mitochondrial diseases. By enhancing mitochondrial priming and stability, FP‐MTs encapsulated in fusogenic liposomes effectively alleviate mitochondrial stress, improve mitochondrial biogenesis, and restore muscle structure and function in bioengineered 3D skeletal muscle models.

The therapeutic potential of FP‐MTs extends beyond muscle regeneration to address mitochondrial dysfunction in conditions such as neurodegenerative diseases (e.g., Parkinson's and Alzheimer's), cardiovascular diseases, and metabolic syndromes. As NADH plays a central role in the electron transport chain and ATP production, NIR‐based NADH uptake and FP‐MT delivery could offer significant benefits for these diseases. Future studies will explore FP‐MT's efficacy in these models and its broader applicability.

Despite its promise, several challenges remain for clinical translation. The limited tissue penetration of NIR light requires innovative solutions, such as optical fiber devices or nanoparticles for localized energy transfer. Additionally, the stability and functionality of FP‐MTs in vivo must be validated, particularly regarding immune responses, retention in target tissues, and long‐term functional integration. Optimization of light parameters and delivery methods will also be crucial to improve systemic and localized therapeutic outcomes.

By addressing these challenges, FP‐MTs could expand their therapeutic applications to treat mitochondrial dysfunction in neurons, cardiomyocytes, and other high‐energy‐demanding cells. For example, FP‐MTs may restore neuronal energy metabolism, delay neurodegeneration, or support cardiomyocyte recovery in cardiac diseases.

In conclusion, this study demonstrates the potential of FP‐MTs and NIR‐mediated NADH delivery to revolutionize mitotherapy‐based treatments. Future research will focus on overcoming current limitations, optimizing delivery systems, and validating FP‐MTs in various disease models to maximize their therapeutic potential.

## 4 Experimental Section

### Materials

Materials for producing FLs have been described previously.^[^ ^14^ ^]^ High‐glucose Dulbecco's modified Eagle's medium (DMEM), fetal bovine serum (FBS), and phosphate‐buffered saline (DPBS) were purchased from HyClone (Utah, USA). Antibiotic‐antimycotic and trypsin–EDTA were purchased from Gibco (MA, USA). β‐Nicotinamide adenine dinucleotide (NADH) was purchased from Roche Diagnostics (Mannheim, Germany). pHrodo Green Dextran (10000 MW) for endocytosis, Hoechst 33342, Nonyl Acridine Orange (Acridine Orange 10‐Nonyl Bromide) (NAO), Mitotracker Green FM, Mitotracker Deep Red FM, tetramethylrhodamine (ethyl ester), perchlorate (TMRE), and SYTO Deep Red Nucleic Acid Stain for live cells were purchased from Invitrogen (CA, USA). The ATP Assay Kit was purchased from BIOMAX (Gurye, Korea), and the CCO Assay Kit and SAA (sarcomeric α‐actinin, ab137346) was purchased from Abcam (Cambridge, UK). Seahorse assay and Seahorse cell culture plates were purchased from Agilent (CA, USA).

### Light Source

The light source used was a constant mode‐LED array driver (LAD‐4) system and an LED array (LEDA4‐x), manufactured by Bio Research Center (Nagoya, Japan). The optical power density (mW cm^−^^2^) was calculated by integrating the obtained spectrum, and light dosages (J/cm^2^) were determined by multiplying the optical power density by the irradiation time. A near‐infrared wavelength of 830 nm was applied, covering an area of ≈1 cm^2^, resulting in an irradiated power density of 10 mW cm^−^^2^. Details of the irradiation setups were described previously.^[^ ^13^ ^]^

### NADH Delivery in L6 Cell

Rat myoblast L6 cells, obtained from ATCC (Virginia, USA), were cultured in high‐glucose DMEM supplemented with FBS and an antibiotic‐antimycotic solution. Cells were maintained in a humidified incubator at 37 °C with 5% CO₂. For NADH delivery studies, prepared cells were exposed to 10 mW cm^−^^2^ of 830 nm near‐infrared (NIR) light for 10 min, delivering an energy density of ≈6 J cm^−^^2^.

Cells were incubated with 20 µm NADH and pH rodo (final concentration, 100 µg mL^−1^) and then exposed to the NIR source to stimulate NADH internalization. Intracellular NADH delivery was monitored using a CLSM, Olympus FV3000, Tokyo, Japan), providing detailed images of NADH uptake.

### Mitochondrial Activity and Energy Metabolism Assays

CCO, TMRE, and ATP analyses were conducted according to established protocols, using CLSM imaging or quantitative measurements.

For the Seahorse assay, L6 cells were seeded at a density of 4.0 × 10⁴ cells per well in Seahorse cell culture plates (103774‐100, Agilent), and the assay was performed following the manufacturer's instructions.

To examine MT morphology within each cell, cells were harvested and fixed with 4% paraformaldehyde (PFA) for 20 min. After fixation, cells were embedded in resin, sectioned, stained with uranyl acetate, washed, and observed by TEM; Hitachi, Tokyo, Japan).

### Mitochondria Isolation

MTs were isolated from L6 cells by differential centrifugation using the Qproteome Mitochondria Isolation Kit (QIAGEN, Hilden, Germany). The MT pellet was resuspended in DPBS at 4 °C. For quantification, the isolated MTs were lysed for 1 min in 2% CHAPS (C3023, Thermo Fisher Scientific) in TBS, and the concentration of the isolated MT was measured using a BCA protein assay kit (23227, Thermo Fisher Scientific).

### Structural and Biogenetic Stability Analysis of Isolated Mitochondria

To evaluate mitochondrial structural stability, isolated mitochondria were stored at 4 °C and assessed daily over a 2‐days period. Each day, 10 µg of mitochondria were stained with Mitotracker, imaged using a CLSM, and visualized as voxel images.

To analyze the lifespan of mitochondria, genomic DNA was extracted from isolated mitochondria. Isolated mitochondria were stored at 4 °C prior to gDNA extraction using a gDNA prep kit (106‐101, GeneAll). PCR was performed using the primers listed in Table S1 (Supporting Information) under appropriate conditions, and the PCR products were visualized using a gel documentation imaging system (ChemiDoc MP, Bio‐Rad Laboratories, Korea).

### Fabrication of Fusogenic Liposome‐Encapsulated Mitochondria and Mitochondrial Uptake Quantification

FLs were synthesized using the film hydration method. Lipid components, including PE, DOTAP, and Liss Rhod PE, were mixed in chloroform at 1:2:0.1 molar ratios. After overnight evaporation of chloroform, the lipids were dispersed in distilled water at 6 mg mL^−1^ and stored at 4°C. Fusogenic liposome‐encapsulated MTs were prepared by mixing isolated MT with liposomes at a 1:2 mass ratio and 1:5 volume ratio through multiple pipetting. Mitochondria were loaded in liposomes at 4 µg per 1.5 × 10⁵ recipient cells.

To quantify MT delivery using the xenograft method, MSC cells were seeded at 3.0 × 10⁵ in a six‐well cell culture plate and 8 µg FMT and FP‐MTs were delivered to MSCs for 1 h. Genomic DNA was extracted from MSCs using a gDNA prep kit. PCR was performed using the primers listed in Table S1 (Supporting Information) under appropriate conditions, and the PCR products were visualized by a gel documentation imaging system.

### RNA Sequencing

Total RNA was extracted from each experimental group (*n* = 3 per group) using the RNA Prep Kit (K‐3140, Bioneer, Korea) according to the manufacturer's instructions. mRNA sequencing was outsourced to eBiogen (Seoul, Korea). The quality of the raw sequencing data was assessed using FastQC to ensure sequencing accuracy. Statistical analysis, including DEG analysis, was performed using the ExDEGA (v5.0.0) tool provided by eBiogen. Genes were considered significantly differentially expressed if they met the threshold of an absolute log2 fold change (|log2FC|) > 1 and an adjusted p‐value < 0.05.

### Western Blotting

Proteins were extracted by lysing samples in ice‐cold radioimmunoprecipitation assay (RIPA) buffer (Thermo Fisher Scientific, Waltham, MA) containing a phosphatase inhibitor cocktail (MilliporeSigma). Protein concentrations were determined using a BCA assay (23225, Thermo Fisher Scientific). A total of 40 µg of protein lysate was resolved on an 8–12% SDS‐PAGE gel and transferred onto a PVDF membrane. After blocking, the membranes were incubated with primary antibodies specific to the target protein, followed by HRP‐conjugated secondary antibodies raised against the host species of the primary antibody. Protein bands were visualized using a gel documentation imaging system.

### qRT‐PCR

qRT‐PCR was performed to assess mRNA levels for each marker. Total RNA was extracted from each group using an RNA prep kit, and cDNA was synthesized with M‐MLV Reverse Transcriptase (28025013, Invitrogen). Real‐time PCR was then conducted using SYBR Green master mix (Takara, Japan) and the primers listed in Table S1 (Supporting Information), with optimized conditions for each primer set.

### Fabrication of Muscle Construct Frame

The muscle structure frame was made of PDMS, with two chambers and a central cylinder fabricated together within a single chip. Specific measurements for fabrication are provided in Figure 4, and the frame was manufactured by the company Microfix (Gyeonggi‐do, Korea) upon request.

### Preparation of Muscle Constructs

The constructs were cultured in 3D hydrogels composed of a fibrinogen and Matrigel mixture, as described previously with some modifications.^[^ ^18f^ ^]^ Briefly, after suspending cells in 12.8 µL growth medium, 3.2 µL Thrombin (Millipore Sigma; 0.1% bovine serum albumin in PBS, 0.2 U mL^−1^), 8 µL Matrigel (Corning), and 16 µL Fibrinogen (Millipore Sigma; 4 mg mL^−1^ in PBS) were added. A 40 µL aliquot of the cell and hydrogel mixture was placed into a sterilized muscle construct frame and allowed to gel for 20 min at 37 °C. Within the hydrogel, 6 × 10⁵ cells and FLs loaded with 16 µg of MT or P‐MT were embedded to induce muscle dysfunction. Muscle constructs were cultured for 2 days in a growth medium containing 1.5 mg mL^−1^ 6‐aminocaproic acid (ACA, Sigma–Aldrich) and 1× antibiotic‐antimycotic. They were then cultured for an additional 26 days in a differentiation medium containing 2 mg mL^−1^ ACA, 2% horse serum, 10 µg mL^−1^ insulin, and 1× antibiotic‐antimycotic to induce myotube formation and construct maturation.

### Measurement of Contractile Forces

The force measurement settings were measured using Curi Bio's (Washington, USA) Mantarray equipment, and NEXEL (Seoul, Korea) was requested to provide service. For measurement, muscle structures were cultured in the Mantarray Stimulation Plate Kit (MA‐1x‐24‐2, Curi Bio), and stimulation was applied at gradually increasing frequencies (1, 2, 3, 5, 10, 20, 30, 40, 50, 60, 70, and 80 Hz) to stimulate the tissue. Twitch force and tetanic force conditions that continuously maintained contraction were confirmed. Twitch and tetanic contraction forces were applied at a stimulation frequency of 0.1 ms at a current of 100 mA. Data analysis was conducted using Microsoft Excel, and a detailed force measurement report was provided by NEXEL, including raw data outputs and analysis of twitch and tetanic contraction forces.

### Muscle Construct Immunostaining

Muscle constructs were washed in PBS and fixed in 2% paraformaldehyde (PFA) overnight at 4 °C, followed by three PBS washes. Muscle constructs were then permeabilized and blocked in PBS containing 0.2% Triton X−100 and 2% bovine serum albumin for 1 h at room temperature. After blocking, samples were incubated with primary antibodies MyoD (sc‐377460, Santa Cruz Biotechnology), Myogenin (sc‐12732, Santa Cruz Biotechnology), and MYH1 (ab127539, Abcam) overnight at 4 °C, and then incubated with the solution containing secondary antibodies and DAPI for 2 h at room temperature. Constructs were placed on a shaker during washing and antibody incubation. Primary and secondary antibodies were diluted in PBS containing 0.2% BSA. Primary antibodies were diluted 200:1 and secondary antibodies were diluted 500:1.

Constructs were cleared for CLSM measurements after passage through a graded methanol series, followed by BABB/methanol and BABB treatment. Briefly, samples were incubated sequentially with 25%, 50%, 75%, 90%, and 100% methanol in PBS for 15 min each at room temperature (RT). For larger samples (>1 mm), incubation times were adjusted as needed. Samples were then transferred to BABB‐resistant plates, treated with a 1:1 BABB/methanol mixture for 30 min at RT, followed by 100% BABB. Samples were fully cleared within 5 min and were either used immediately for analysis or stored in the dark at 4 °C.

To prepare muscle construct sections for immunostaining, PFA‐fixed constructs were incubated overnight in 20% sucrose (MilliporeSigma) and then submerged in optimal cutting temperature compound (Fisher Scientific, Hampton, NH) and frozen at −80 °C for 1 day. Constructs were then sectioned transversely using a Leica Cryostat CM3050S microtome. All samples were imaged using a CLSM, and image analysis was performed using CellSens software.

### Statistical Analysis

Statistical analyses were conducted using GraphPad Prism 8.0.1 software. All data are presented as the mean ± standard deviation (SD). The statistical significance of differences among multiple groups was assessed using one‐way ANOVA followed by Tukey's multiple comparisons test. For comparisons between the two groups, an unpaired t‐test was performed after confirming the normality of the data distribution. Statistical significance was defined as follows: n.s. (not significant), ^*^ *p* < 0.05, ^**^ *p* < 0.01, ^***^ *p* < 0.001, and ^****^ *p* < 0.0001. The sample size (*n*) and detailed statistical tests applied are specified in the corresponding figure legends.

## Conflict of Interest

The authors declare no conflict of interest.

## Author Contributions

K.‐H.P. and H.J.K. acquired funds, performed conceptualization, methodology, and wrote the original draft. H.B.C. and H.J.K. wrote, reviewed, and edited the final draft and curated data. H.B.C. performed project administration, visualization, validation, investigation, and wrote the original draft. H.‐R.K. and S.J. performed investigation, visualization, methodology, and curated data. C.W.C. and J.‐I.P. performed formal analysis and curated data. S.Y., G.S., and S.K. performed visualization and acquired resources. The schematic was adapted from a poster provided by Curibio. Curi Bio & NEXEL: Image copyright and permission for Figure 6 use.

## Supporting information



Supporting Information

## Data Availability

The data that support the findings of this study are available in the supplementary material of this article.
