# Supplementary material for: Near Infrared‐Mediated Intracellular NADH Delivery Strengthens Mitochondrial Function and Stability in Muscle Dysfunction Model
Source: Adv Sci (Weinh). 2025 Jan 31;12(12):2415303. doi: 10.1002/advs.202415303 (PMC11948086; doi:10.1002/advs.202415303)
Supplement: Supplementary file 1 — Supporting Information [file ADVS-12-2415303-s001.docx]

**Near Infrared (NIR)-Mediated Intracellular NADH Delivery Strengthens Mitochondrial Function and Stability in Muscle Dysfunction Model**

**Hui Bang Cho, Hye-Ryoung Kim, Sujeong Lee, Chae Won Cho, Ji-In Park, Seulki Youn, Gyuwon So, Sumin Kang, Hye Jin Kim^*^, and Keun-Hong Park^*^**

Department of Nano-regenerative Medical Engineering, College of Life Science, CHA University, 6F, CHA Biocomplex, Sampyeong-Dong, Bundang-gu, Seongnam-si, 13488, Republic of Korea.

[*] Corresponding authors

Hye Jin Kim; khye4680@naver.com

Keun-Hong Park; pkh0410@cha.ac.kr

**Keywords**

**NADH delivery, Mitochondrial priming, Mitotherapy, Near-infrared (NIR), Muscle regeneration**

**
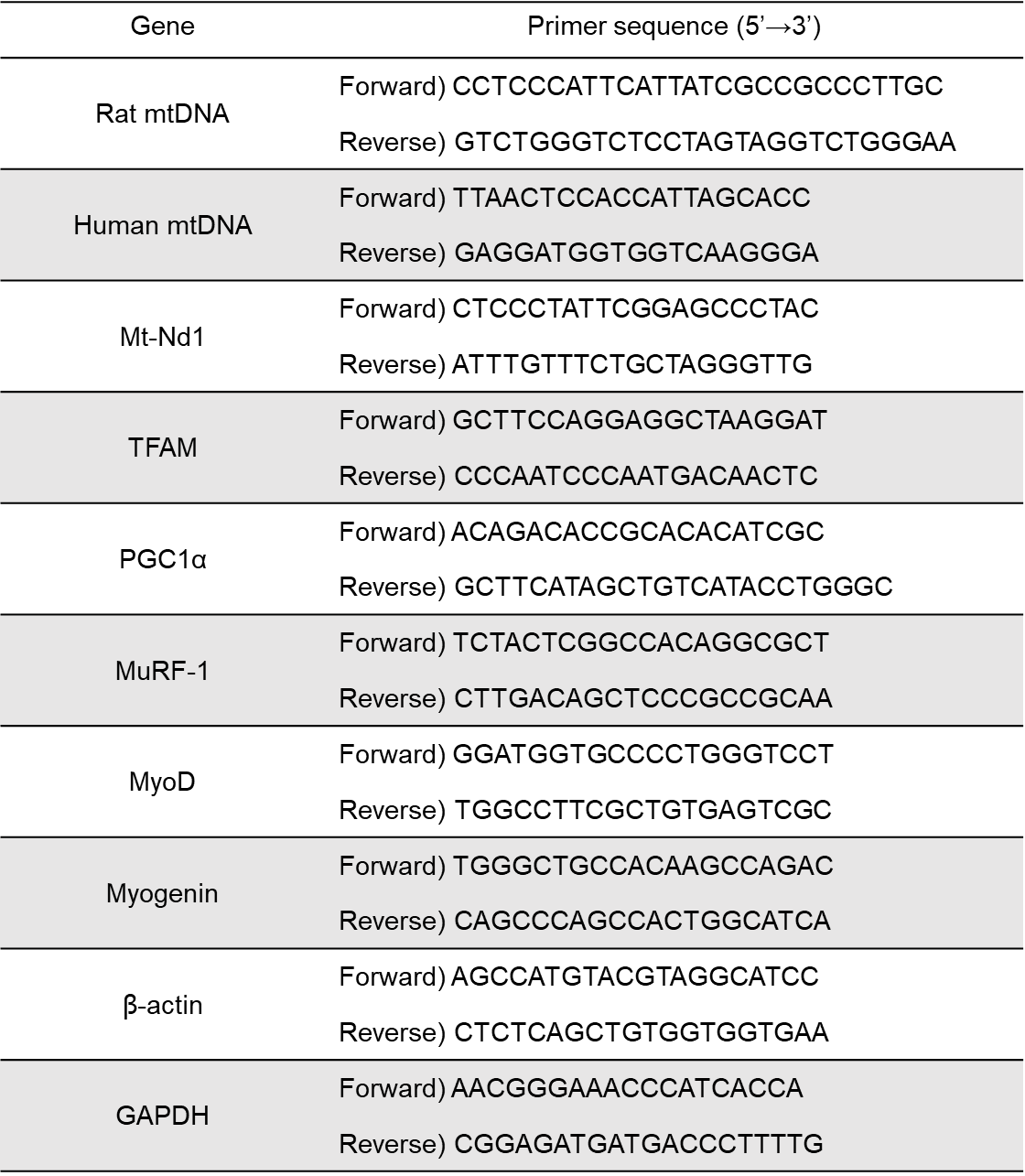
**

**Table S1. qRT-PCR primer sequences.**

**
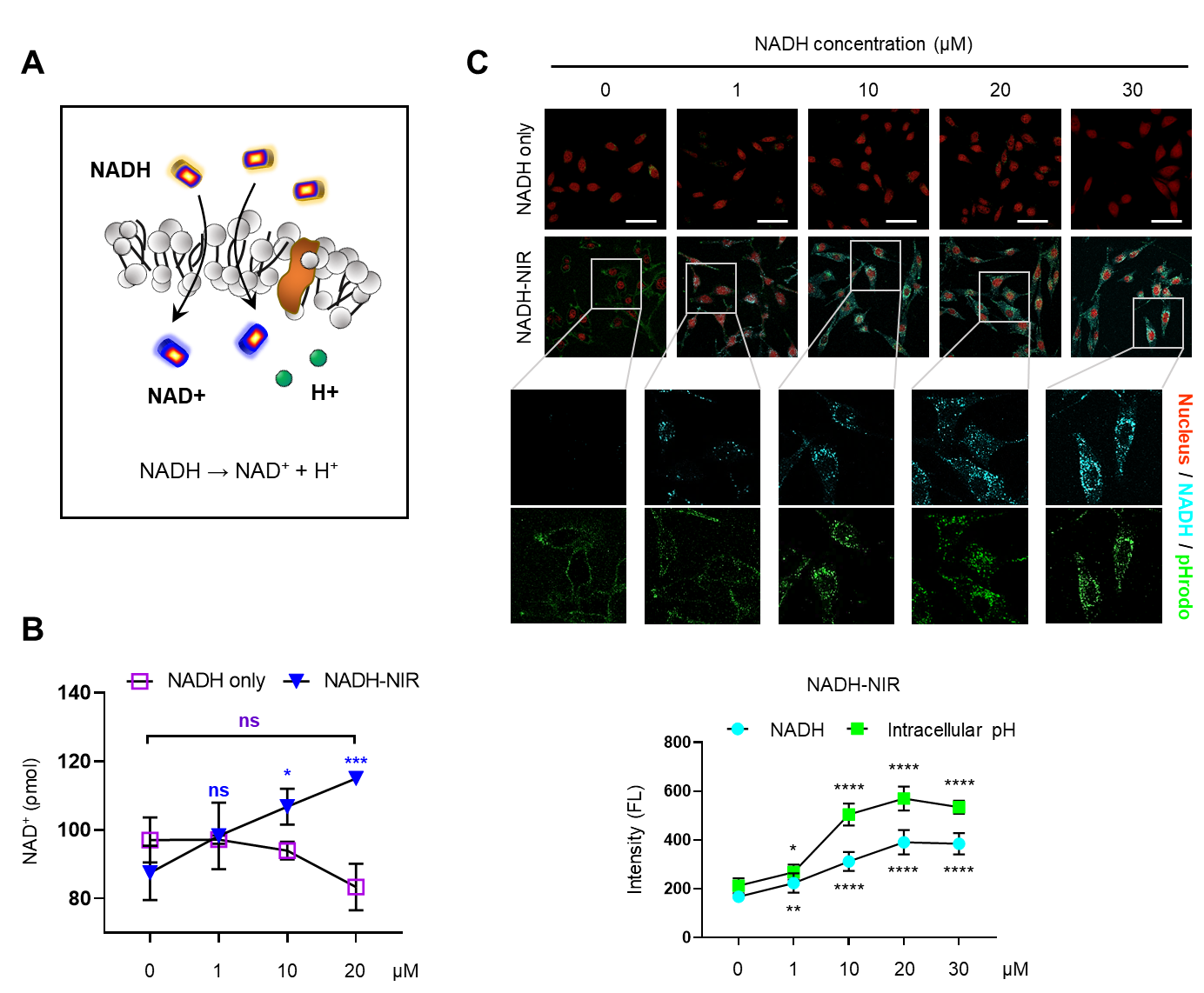
**

**Figure S1. Determination of optimal NADH concentrations for enhanced NADH delivery.**

(A) Schematic illustrating the oxidation of NADH to NAD+ and H+ in L6 cells. (B) Quantification of intracellular NADH levels across different treatment conditions and concentrations (0, 1, 10, 20, and 30 μM) (n=3). (C) Representative confocal microscopy images of intracellular NADH levels across different treatment conditions and concentrations. NADH (cyan), and pH changes (green). Nuclei are shown in red (n=3). Scale bar = 50 μm. Data represent the mean ± SD. Statistical significance was evaluated using one-way ANOVA with Tukey’s multiple comparisons test. ns, not significant. *p < 0.05, **p < 0.01, ***p < 0.001 and ****p < 0.0001.


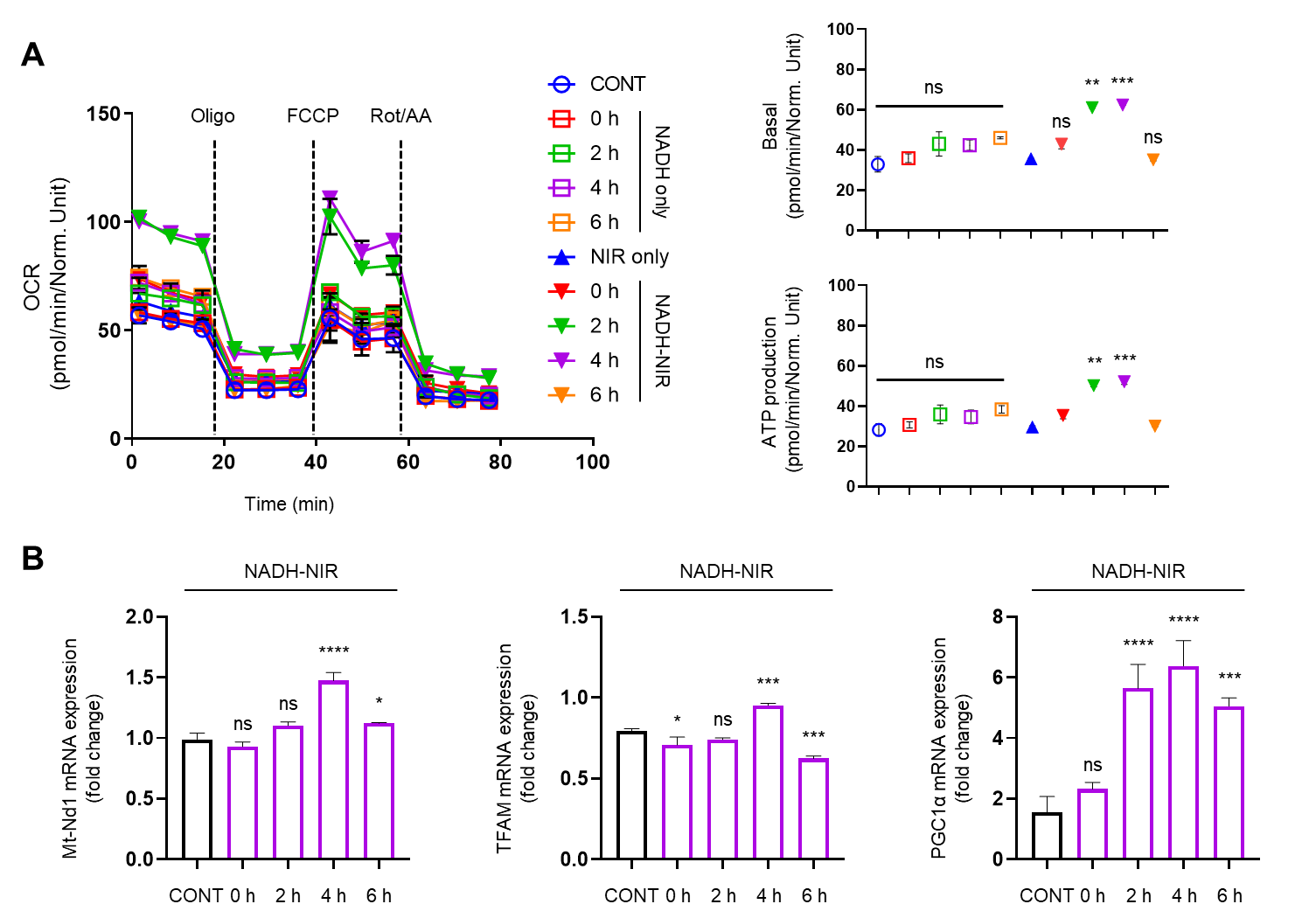


**Figure S2. Optimization of NADH treatment duration for mitochondrial function enhancement.**

Oxygen consumption rate (n=3) (A) and qRT-PCR analysis (n=3) (B) were performed at 0, 2, 4, and 6 h after treatment with 20 μM NADH. Data represent the mean ± SD. Statistical significance was evaluated using one-way ANOVA with Tukey’s multiple comparisons test. ns, not significant. *p < 0.05, **p < 0.01, ***p < 0.001, and ****p < 0.0001.

**
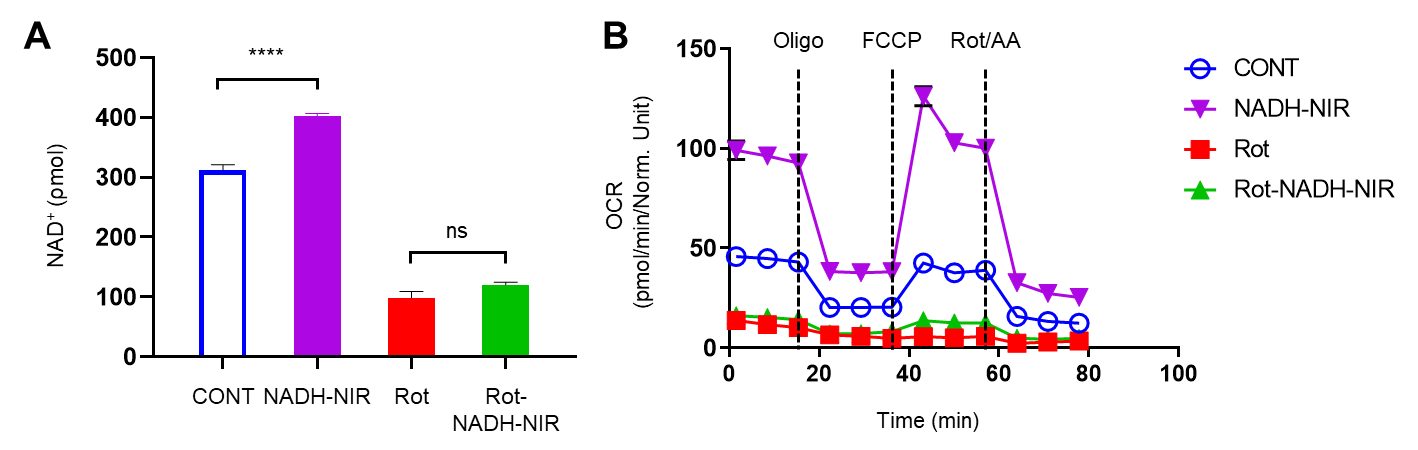
**

**Figure S3. Validation of mitochondrial priming via complex I inhibition.**

NAD+/NADH assay (n=3) (A) and oxygen consumption rate measurements (n=3) (B) were performed following complex I inhibition with rotenone. Data represent the mean ± SD and comparison with the NADH-only group at each time point. Statistical significance was evaluated using one-way ANOVA with Tukey’s multiple comparisons test. ns, not significant and ****p < 0.0001.

**
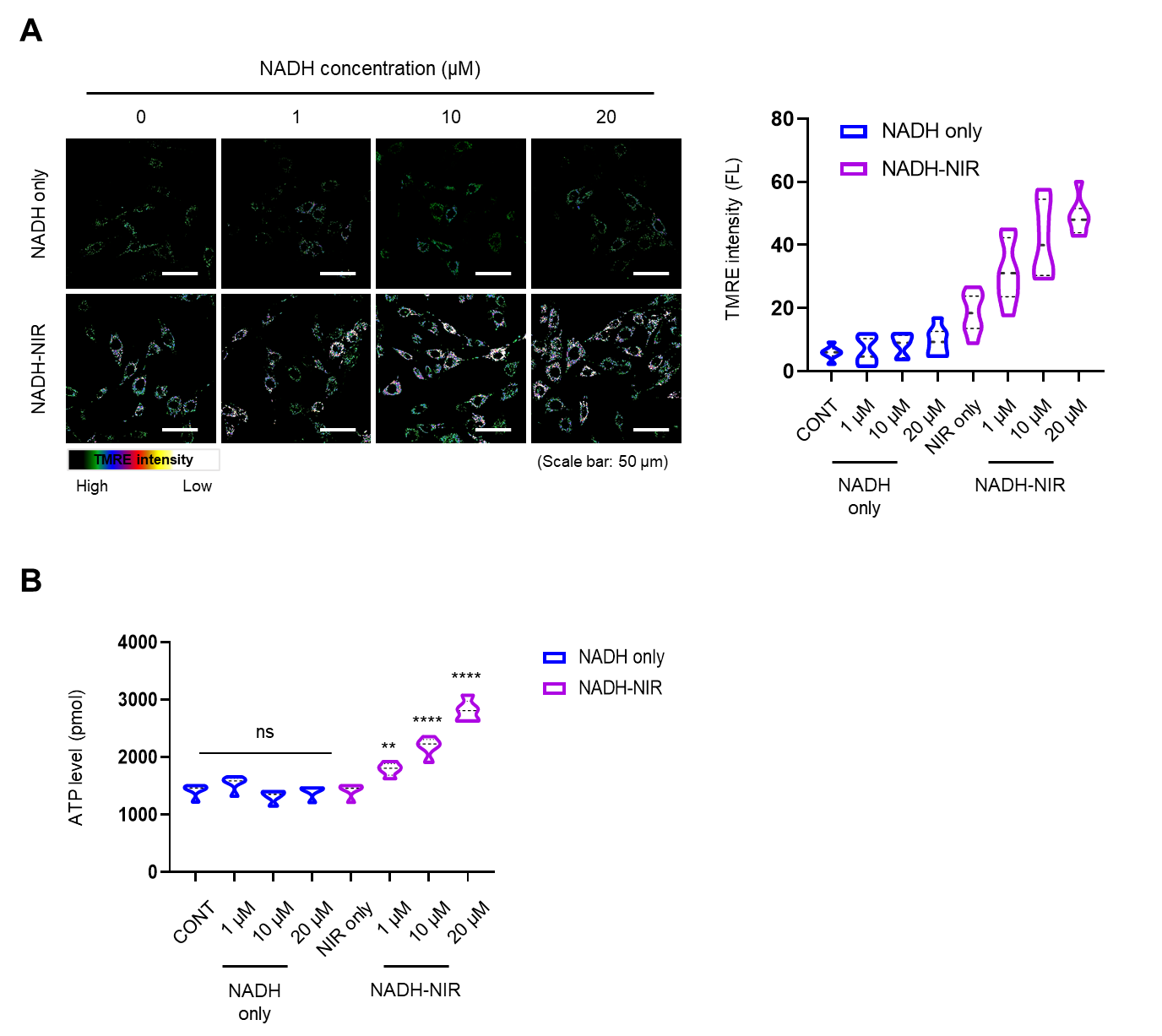
**

**Figure S4. MMP and ATP production after 4-hour mitochondrial priming.**

Representative images of MMP measured using TMRE staining (n=7) (A), along with ATP production levels (n=3) (B). Scale bar = 50 μm. Data represent the mean ± SD and comparison with the NADH-only group at each time point. Statistical significance was evaluated using one-way ANOVA with Tukey’s multiple comparisons test. ns, not significant, **p < 0.01 and ****p < 0.0001.

**
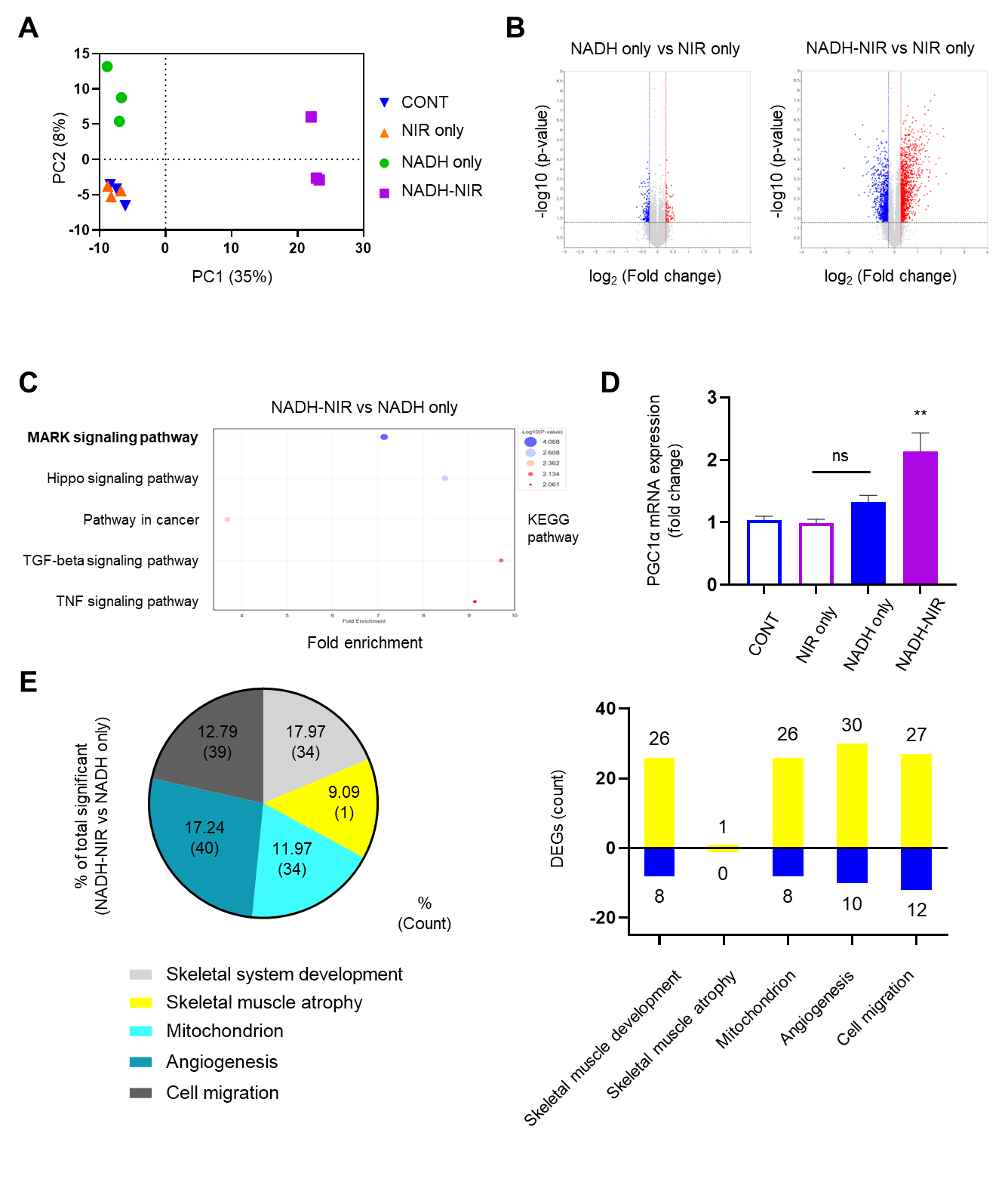
**

**Figure S5. Differential gene expression analysis after NADH-NIR treatment.**

KEGG pathway PCA plot (n=3) (A), Volcano plot (B) KEGG pathway analysis based on the DEG results (C) qRT-PCR (n=3) (D) and GO functional analysis of DEGs showing transcriptome differences (E). DEGs were defined by |log2FC| $>$1 and p value $<$0.05. Statistical significance was evaluated using one-way ANOVA with Tukey’s multiple comparisons test. ns, not significant, **p < 0.01.

**
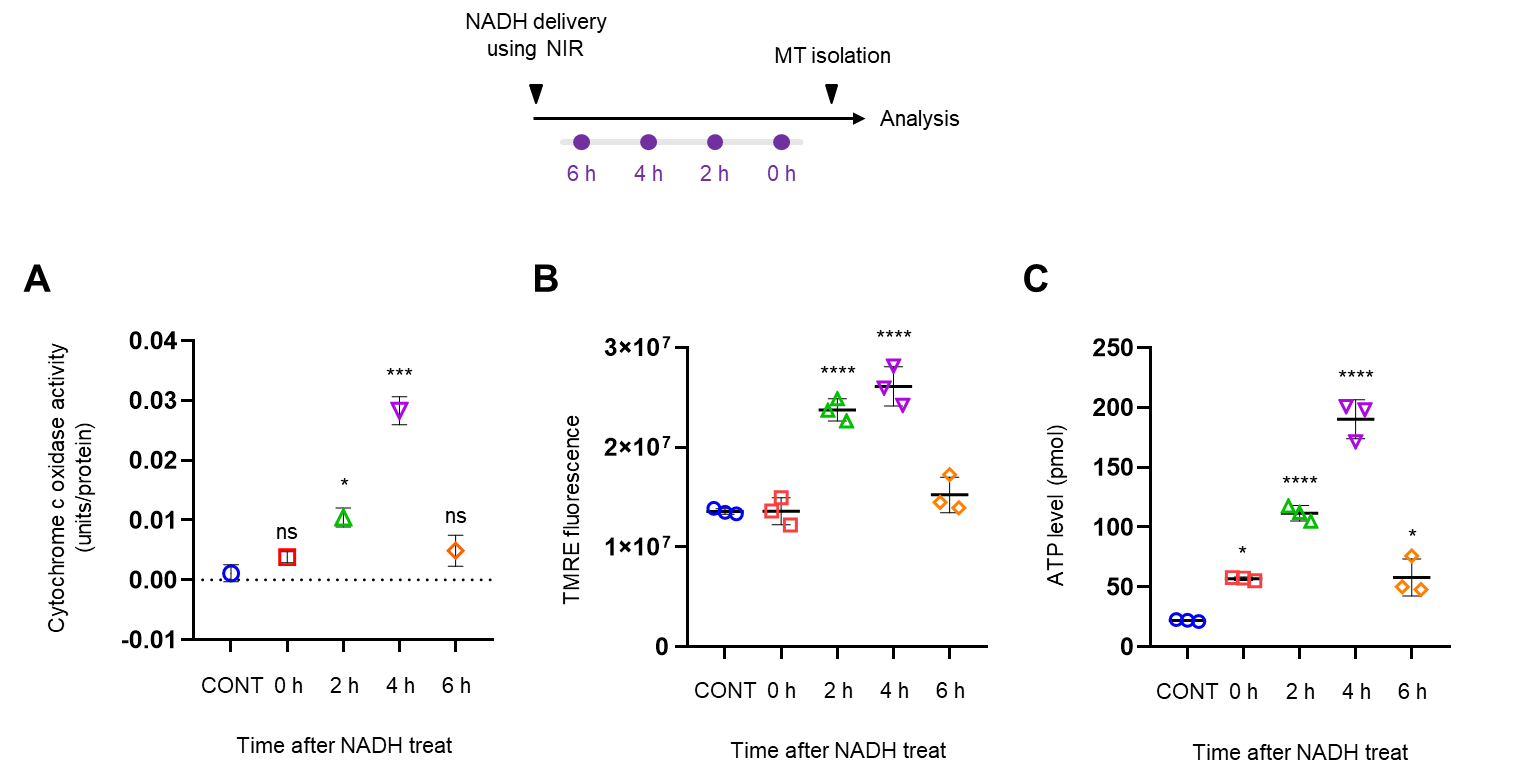
**

**Figure S6. Functional assessment of mitochondria after NADH-NIR.**

Measurements of CCO activity (n=3) (A), TMRE intensity (n=3) (B), and ATP levels (n=3) (C). Statistical significance was evaluated using one-way ANOVA with Tukey’s multiple comparisons test. ns, not significant, *p < 0.05, ***p < 0.001 and ****p < 0.0001.

**
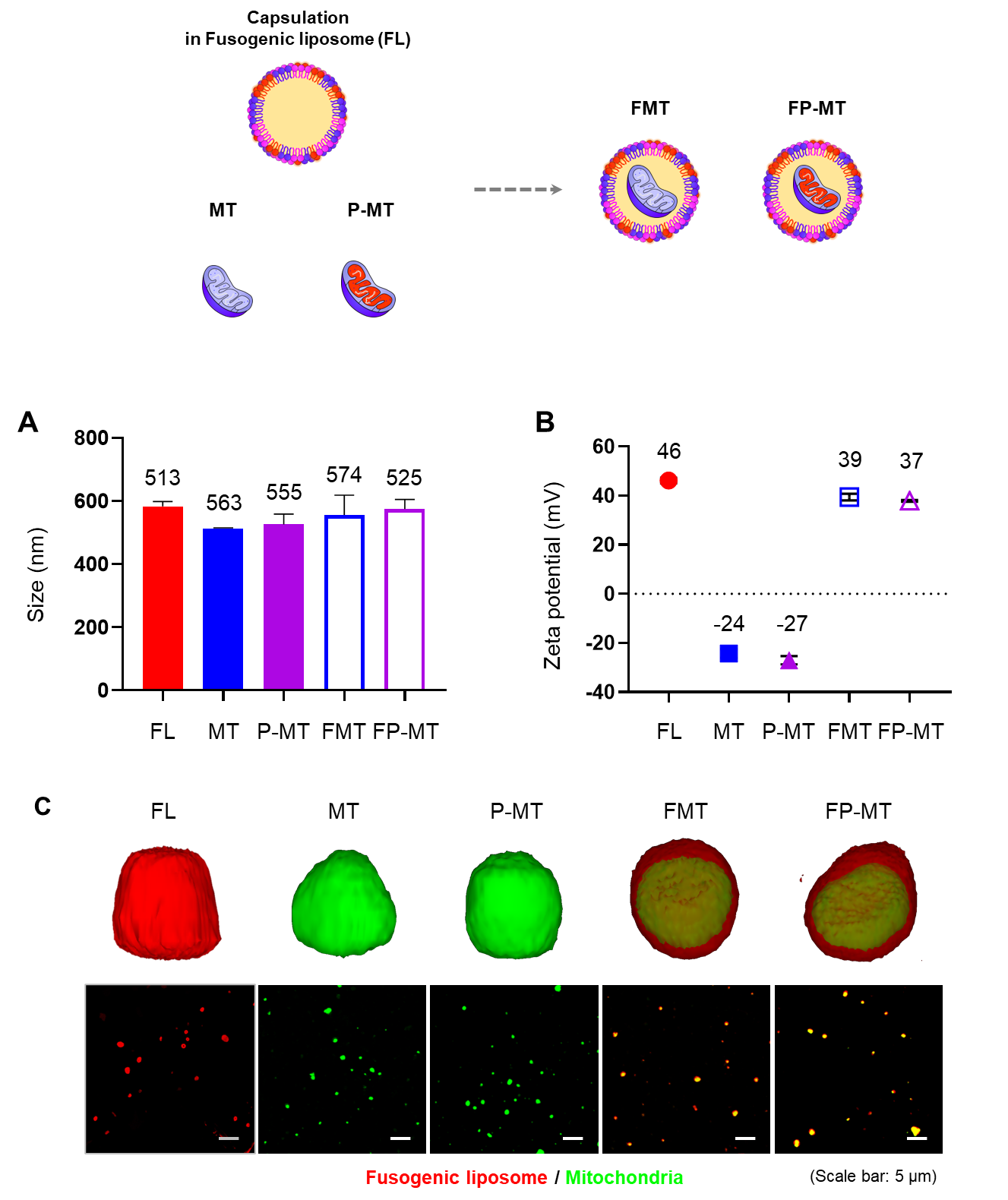
**

**Figure S7. DLS analysis of size (n=3) (A) zeta potential (n=3) (B) and CLSM and voxel images (C) showing FMT and FP-MT. FL (red) and mitochondria (green) are indicated.** Scale bar = 5 μm.


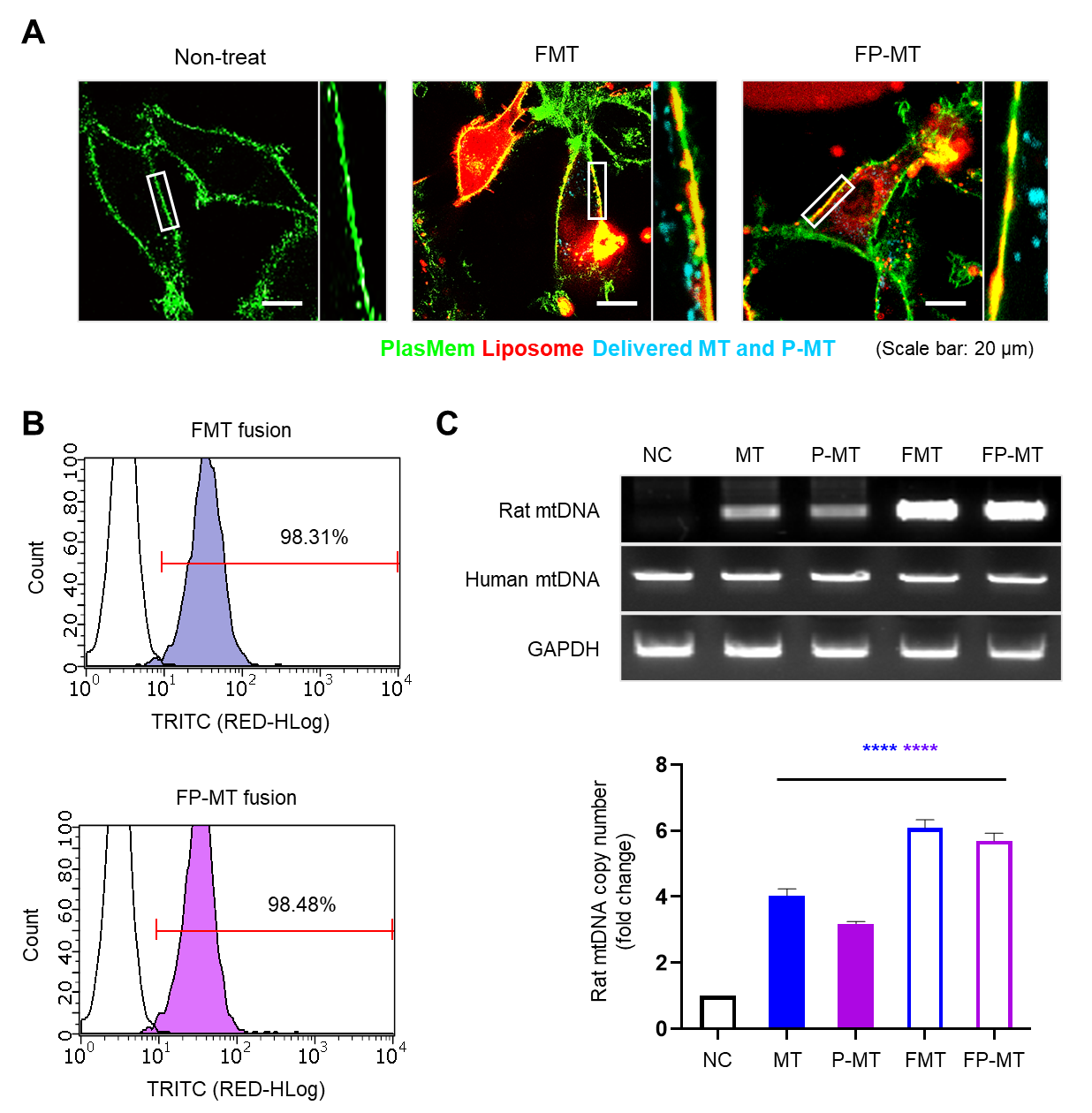


**Figure S8. Internalization pattern and delivery efficiency of FMT and FP-MT.**

(A) Confocal laser scanning microscopy (CLSM) images showing the internalization pattern of FMT and FP-MT within recipient cells, with plasma membrane (green), fusogenic liposome (red), and delivered mitochondria (blue) indicated. Scale bar = 50 μm. (B) Quantification of fusion events by flow cytometry analysis and (C) quantification of delivered mitochondria by mtDNA copy number (n=3). Statistical significance was evaluated using one-way ANOVA with Tukey’s multiple comparisons test. ns, not significant, ****p < 0.0001.


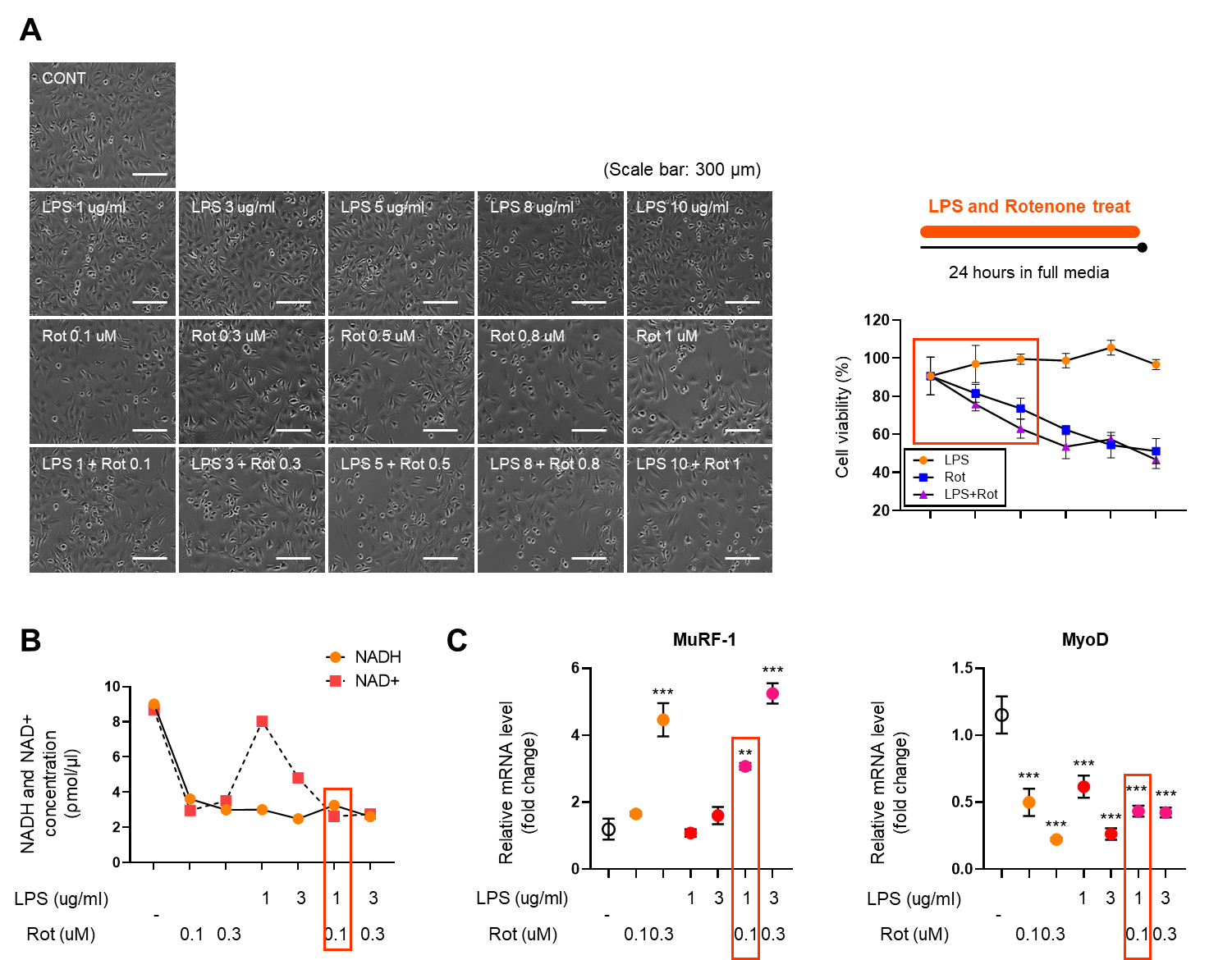


**Figure S9. Optimization of LPS and rotenone co-treatment concentration for modeling muscle dysfunction cells.**

Imaging and WST-1 (n=3) (A), NAD+/NADH assay (n=3) (B), and qRT-PCR analysis (n=3) (C). Scale bar = 50 μm.

**
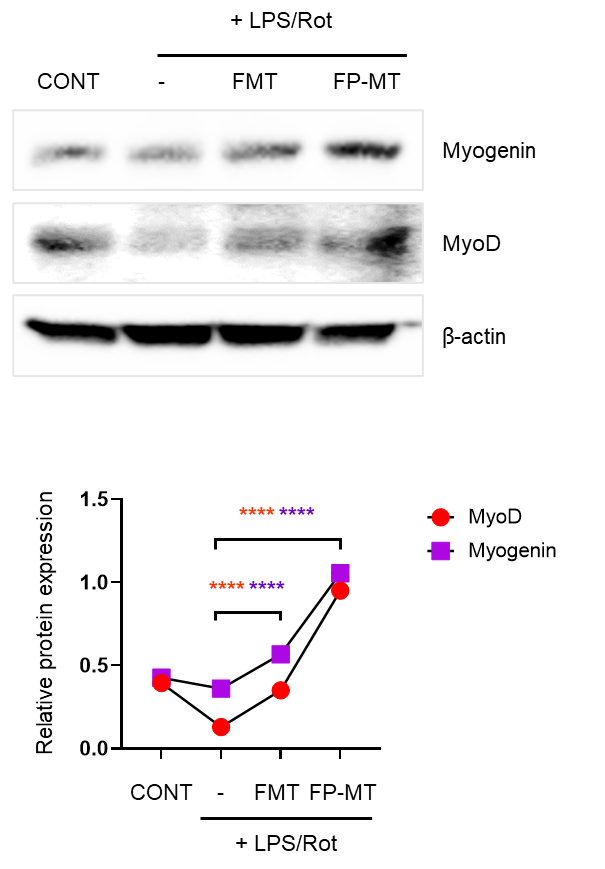
**

**Figure S10. Western blot analysis of myogenesis-related protein expression in response to muscle dysfunction in a 2D muscle dysfunction model (n=3).** Statistical significance was evaluated using one-way ANOVA with Tukey’s multiple comparisons test. ****p < 0.0001.

**
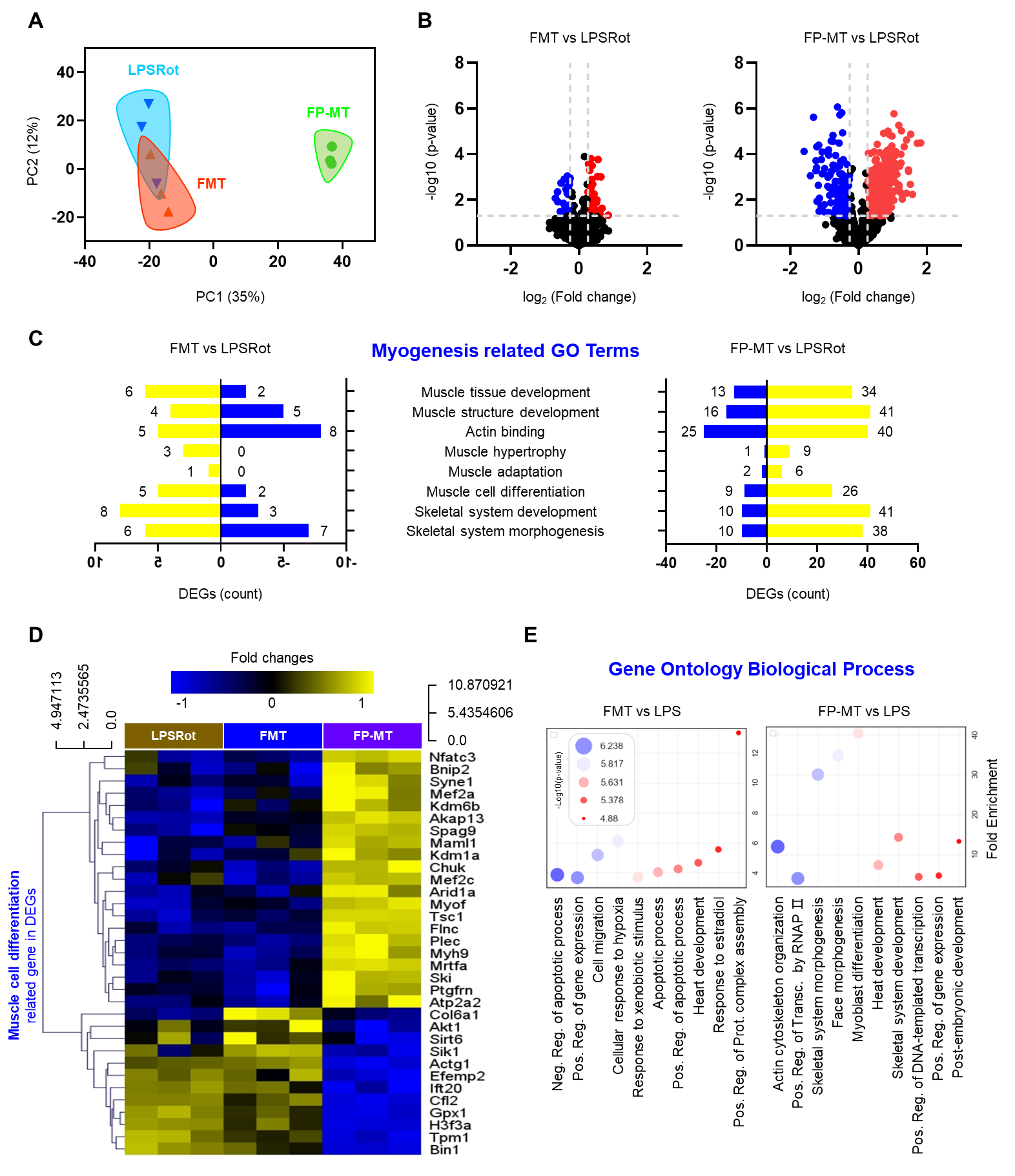
**

**Figure S11. Comparative gene expression and biological process analysis in FMT (n=3) vs. LPS/Rot (n=3) and FP-MT (n=3) vs. LPS/Rot muscle constructs.**

(A) PCA plot illustrating the separation of gene expression profiles of LPS/Rot, MT, and P-MT (B) Volcano plots of differentially expressed genes (DEGs) between MT and LPS/Rot (left) and between P-MT and LPS/Rot (right), showing significant upregulated (red) and downregulated (blue) genes. (C) GO term enrichment analysis for myogenesis-related processes. (D) Heatmap of muscle cell differentiation-related DEGs. (E) GO-BP analysis of DEGs between FP-MT- and FMT-exposed muscle constructs, with bubble plots showing processes. DEGs were defined by |log2FC| $>$1 and p value $<$0.05.

**
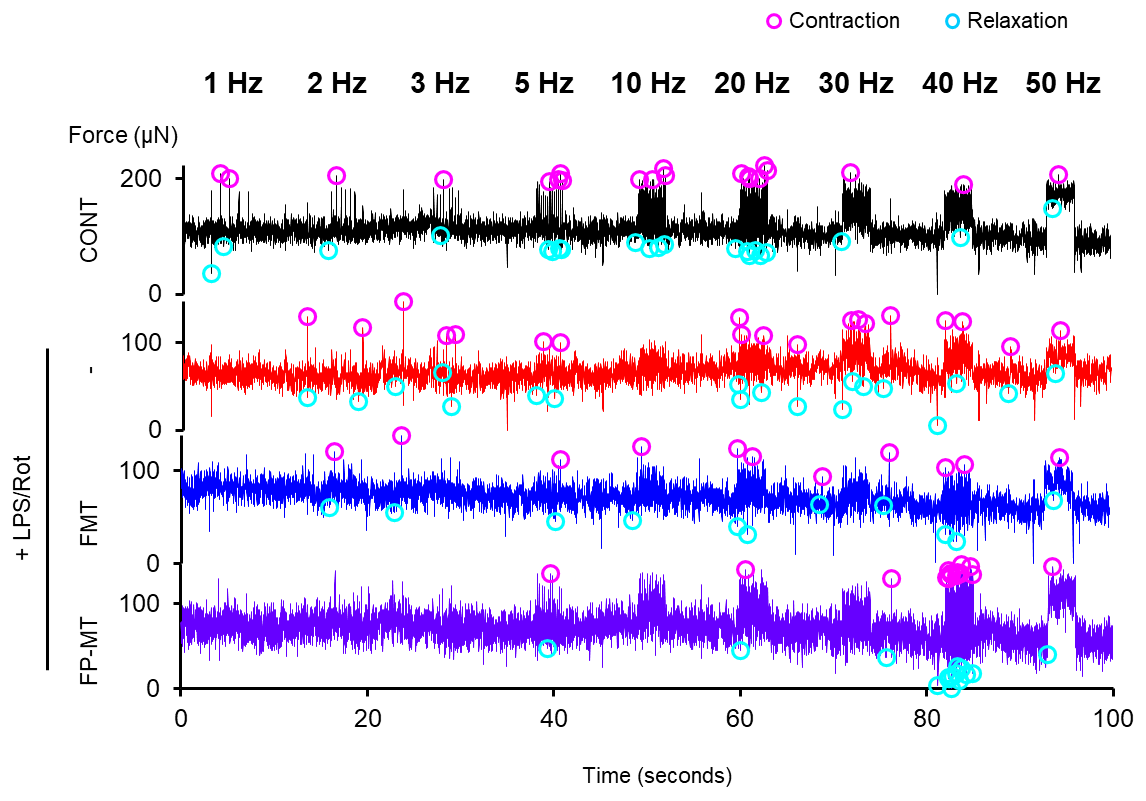
**

**Figure S12. Preliminary screening of tetanic force responses at stimulation frequencies of 1, 2, 3, 5, 10, 20, 30, 40, 50 Hz.**
